# Differential gene expression among three sex types reveals a MALE STERILITY 1 (*CpMS1*) for sex differentiation in papaya

**DOI:** 10.1186/s12870-019-2169-0

**Published:** 2019-12-09

**Authors:** Dessireé Zerpa-Catanho, Jennifer Wai, Ming Li Wang, Li’ang Yu, Julie Nguyen, Ray Ming

**Affiliations:** 1 Department of Plant Biology, University of Illinois at Urbana-Champaign, Urbana, IL 61801 USA; 2 Hawaii Agriculture Research Center, Kunia, HI 96759 USA

**Keywords:** *Carica papaya*, Flower development, Male sterility gene, RT-qPCR, RNA-Seq, Sex differentiation

## Abstract

**Background:**

Carica papaya is a trioecious plant species with a genetic sex-determination system defined by sex chromosomes. Under unfavorable environmental conditions male and hermaphrodite exhibit sex-reversal. Previous genomic research revealed few candidate genes for sex differentiation in this species. Nevertheless, more analysis is still needed to identify the mechanism responsible for sex flower organ development in papaya.

**Results:**

The aim of this study was to identify differentially expressed genes among male, female and hermaphrodite flowers in papaya during early (pre-meiosis) and later (post-meiosis) stages of flower development. RNA-seq was used to evaluate the expression of differentially expressed genes and RT-qPCR was used to verify the results. Putative functions of these genes were analyzed based on their homology with orthologs in other plant species and their expression patterns. We identified a Male Sterility 1 gene (*CpMS1*) highly up-regulated in male and hermaphrodite flower buds compared to female flower buds, which expresses in small male flower buds (3–8 mm), and that might be playing an important role in male flower organ development due to its homology to MS1 genes previously identified in other plants. This is the first study in which the sex-biased expression of genes related to tapetum development in the anther developmental pathway is being reported in papaya. Besides important transcription factors related to flower organ development and flowering time regulation, we identified differential expression of genes that are known to participate in ABA, ROS and auxin signaling pathways (ABA-8-hydroxylases, AIL5, UPBEAT 1, VAN3-binding protein).

**Conclusions:**

*CpMS1* was expressed in papaya male and hermaphrodite flowers at early stages, suggesting that this gene might participate in male flower organ development processes, nevertheless, this gene cannot be considered a sex-determination gene. Due to its homology with other plant MS1 proteins and its expression pattern, we hypothesize that this gene participates in anther development processes, like tapetum and pollen development, downstream gender specification. Further gene functional characterization studies in papaya are required to confirm this hypothesis. The role of ABA and ROS signaling pathways in papaya flower development needs to be further explored as well.

## Background

Unisexual flowers in angiosperm plant species are classified as monoecious or dioecious. Monoecious plant species have female and male flowers in separate flowers but on the same individual (6% angiosperm species), while dioecious species have female and male flowers on separate individuals (5% angiosperm species). Dioecious plant species could evolve from hermaphroditic or monoecious populations in three major steps [1, 2]. First, a recessive male-sterile mutation occurred originating female plants. The occurrence of this mutation would be advantageous for the population, because female plants could be pollinated by individuals from different populations, reducing the inbreeding and increasing the genetic variability within the population. Later, a second dominant female-sterile mutation appeared in the monoecious population generating male plants. With time, the pair of chromosomes in which these mutations occurred stopped recombining and started accumulating mutations and repetitive elements. Recombination stopped because an individual with both mutations would become completely sterile, representing no advantage for the population. Finally, the chromosomes carrying these mutations became a pair of different sex chromosomes [1, 2].

Sex chromosomes are one of the most studied systems for sex determination in dioecious plants, and there are several stages of sex chromosomes already identified in many plant species [2, 3]. Some of these species have been considered as important models for the study of sex determination and sex chromosome evolution in dioecious plants, including papaya (*Carica papaya*) [4]. Nevertheless, papaya is considered a trioecious species, which means that papaya populations can have individuals with either male, female or hermaphrodite flowers [5]. Natural papaya populations are dioecious, while cultivated populations are gynodioecious. In papaya, sex is determined by a recent sex chromosome system with three different sex chromosomes (X, Y, and Y^h^). Female plants are homozygous for the X chromosome (XX) and males and hermaphrodites are heterozygous (XY and XY^h^, respectively) [6, 7]. Between the X and Y^h^ chromosomes, several differences have been identified that can explain phenotypic differences between hermaphrodite and female plants [7]. In contrast, the Y and Y^h^ chromosomes have been described as highly similar (99.60%) [6, 8] and as a result, it has been challenging to explain which differences observed between male and hermaphrodite plants are responsible for their phenotypes.

Despite the genetic differences found among all these three sex chromosomes, flower development among papaya plants is very similar in its early stages. Male, female and hermaphrodite flower development start to differentiate after anthers develop in male and hermaphrodite flowers [9, 10]. One of the main differences among the flowers is the presence of a gynoecium spear-like structure called ‘pistillode’ (or rudimentary pistil) in male flowers instead of a functional gynoecium, like in hermaphrodite and female flowers [9, 10]. For this reason, it is believed that a female-sterile dominant mutation suppresses the carpel development in male flowers and that this mutation exists on the Y chromosome, but not on the Y^h^ chromosome. Since the Y and the Y^h^ chromosome are highly similar and most of the detected genetic differences or mutations are located on introns instead of exons [6, 8], differential gynoecium development in hermaphrodite plants and not in male plants is believed to be the result of the differential expression of a carpel development suppressor gene between sex-types. Likewise, female flowers do not have stamens, but male and hermaphrodite flowers do [9, 10]. Therefore, a gene with male-promoting functions is believed to be located on the Y and the Y^h^ chromosome.

An interesting aspect of papaya plants is that under certain environmental conditions or stimuli (e.g. high or cold temperatures, shorter day length, water stress, and terminal bud injury) male and hermaphrodite plants can switch their flower gender [11–16]. This phenomenon is known as sex-reversal and evidently affects papaya fruit production, because under undesirable environmental conditions, hermaphrodites could either reverse to male or present staminal carpellody (a condition in which the stamen resemble carpel or are ‘fused’ to the carpels), which results in malformed unmarketable papaya fruits [10, 17–19]. Interestingly female plants do not suffer sex-reversal, as male and hermaphrodites do. Therefore, identifying the genes responsible for the correct expression of sex or development of sex flower organs in papaya and the regulatory mechanism for the expression of those genes becomes fundamental for papaya production.

To identify the genes responsible for the correct expression of sex in papaya flowers, previous researchers have looked at the expression of homeotic genes that participate in the ABC model for flower development. There are few reports about differentially expressed genes among sex types and on flower development regulation by MADS-box genes in papaya [16, 20–24]. Recently, a digital transcriptome analysis of the genes located on the X and Y^h^ chromosomes in papaya using high-throughput SuperSAGE technique combined with a whole-genome sequence comparison between male and hermaphrodite plants identified a Short Vegetative Phase (SVP) gene and a Monodehydroascorbate Reductase (MDAR) gene as candidates for sex determination in papaya [23, 25]. Furthermore, a recent transcriptome analysis using RNA-sequencing has suggested the silencing of the carpel suppression function by epigenetic modifications (miRNAs) in male-to-hermaphrodite induced sex reversal plants [16]. A recent study, proposed three candidate sex-related loci, including the Short Vegetative Phase (SVP) gene and a Chromatin Assembly Factor 1 subunit A-like (CAF1AL), as responsible for regulating correct flower development in papaya, based on alternative splicing and differential expression analysis using different flower whorls [26]. Nevertheless, there is no published comparative transcriptome analysis focused on different developmental flowering stages using RNA-sequencing in papaya, including all three different sex types (including male, female and hermaphrodite flowers). Therefore, further analysis is still needed to identify the mechanisms responsible for flower development regulation in papaya, carpel development suppression in male flowers, stamen carpellody in hermaphrodite flowers and the sex reversal phenomena that occurs only in male and hermaphrodite papaya flowers.

RNA sequencing or RNA-Seq consists of the implementation of high-throughput DNA sequencing technologies for the study of transcriptomes [27, 28]. RNA-Seq has been described as a very powerful tool for the discovery of novel transcripts and the quantification of gene expression in model and non-model plant species, which ultimately leads to the identification of differentially expressed genes, pathways and regulatory networks that help to understand biological processes. Therefore, a differential gene expression analysis of flower buds among all three different sex types at different developmental stages during flowering can help to find differentially expressed genes associated with correct sex expression, as well as to better understand flower organ development regulation in papaya. The aim of this study is to identify genes that are differentially expressed among male, female and hermaphrodite flower buds in papaya during early and later stages of flower development using RNA-seq, and to evaluate the expression of highly differentially expressed genes by RT-qPCR, as well as to identify the putative functions for these genes based on their homology with other plant species and their expression patterns.

## Results

### Quality control before RNA-Seq and differential expression analysis

The transcriptome of papaya flower buds from male ‘AU9’, female ‘AU9’ and hermaphrodite ‘SunUp’ plants was sequenced at two different developmental stages (pre-meiosis: 1–6 mm and post-meiosis: 7–12 mm) (Additional file 7: Table S1). On average, a total of 2.28E+ 07 raw reads per library were obtained (Additional file 7: Table S1). In general, the quality of the raw reads was classified as good by the FastQC program. Nevertheless, after trimming low-quality reads and adaptors, an average of 99.71% of these raw reads with an average length of 100 bp remained. These high-quality reads were aligned to the papaya genome. On average, a total of 83.99% reads per library were aligned uniquely to the genome, and few reads were not aligned or aligned more than once to the genome (Additional file 7: Table S1). On average, 46.08% of the reads that aligned to the genome were assigned to exons (Additional file 7: Table S1). After normalization of the reads and before the differential expression analysis, samples were clustered, and the biological coefficient of variation was calculated as part of our analysis of quality control (Additional file 1: Figure S1). Samples clustered in three groups, one group composed of normal and teratological males of the variety ‘Zhonghuang’, a second group composed of female ‘AU9’ samples, and the third group composed by male ‘AU9’ and hermaphrodite ‘SunUp’ samples. These results reflect the existence of fewer differences found between female pre-meiosis and female post-meiosis stages, and fewer differences between male and hermaphrodite pre-meiosis stages than post-meiosis stages. No confounding batch effect was found and the calculated trend for the Biological coefficient of variation was not far from the calculated common trend (Additional file 1: Figure S1). Therefore, the analysis of differentially expressed genes was performed using the normalized expression values.

### Differential gene expression analysis by RNA-Seq

**Fig. 1:**
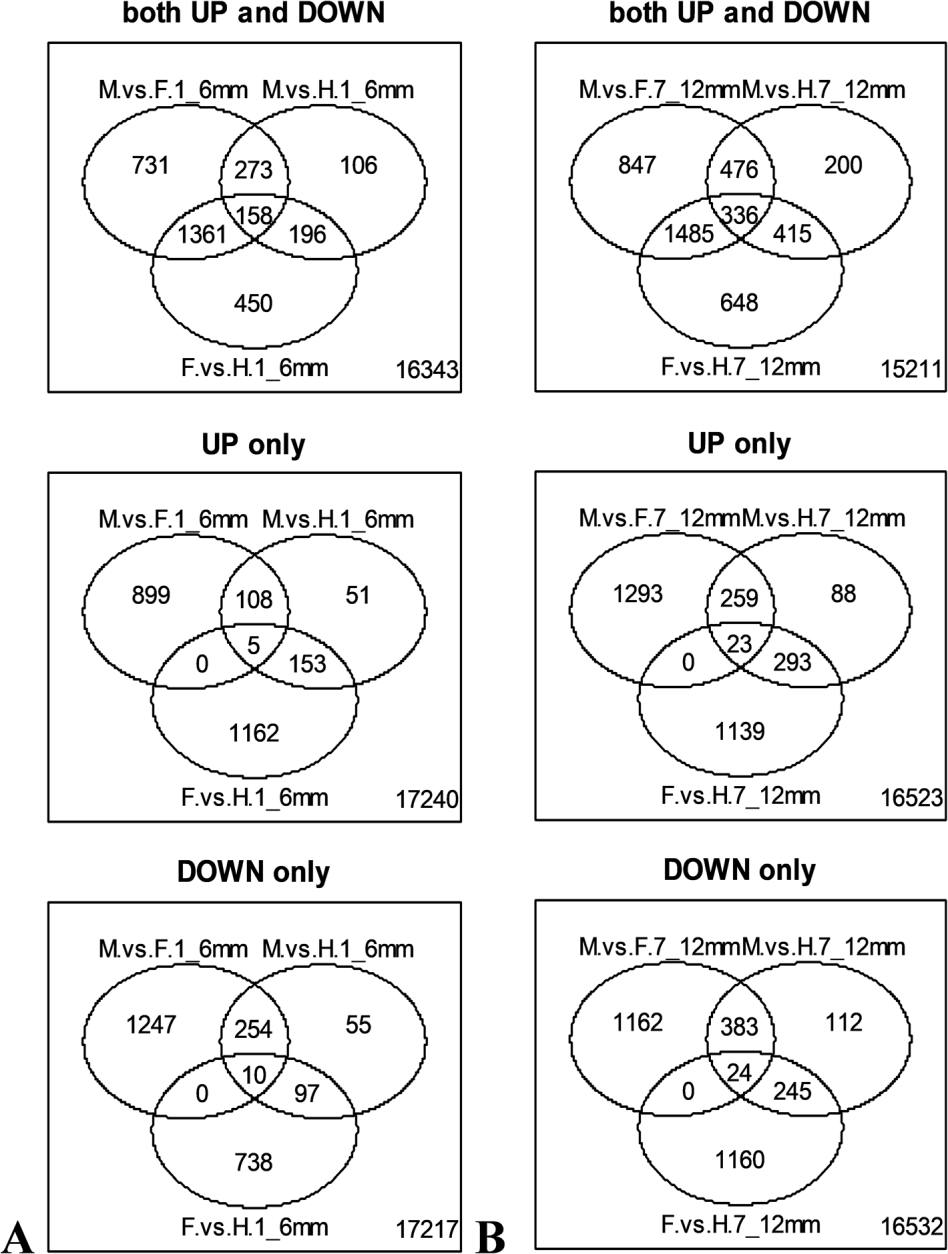
Venn diagrams showing the number of differentially expressed genes (up and down-regulated, only up-regulated or only down-regulated) between male, female and hermaphrodite flower buds of different sizes (**a**. flower buds size: 1-6 mm, **b**. flower buds size: 7-12 mm)

**Fig. 2:**
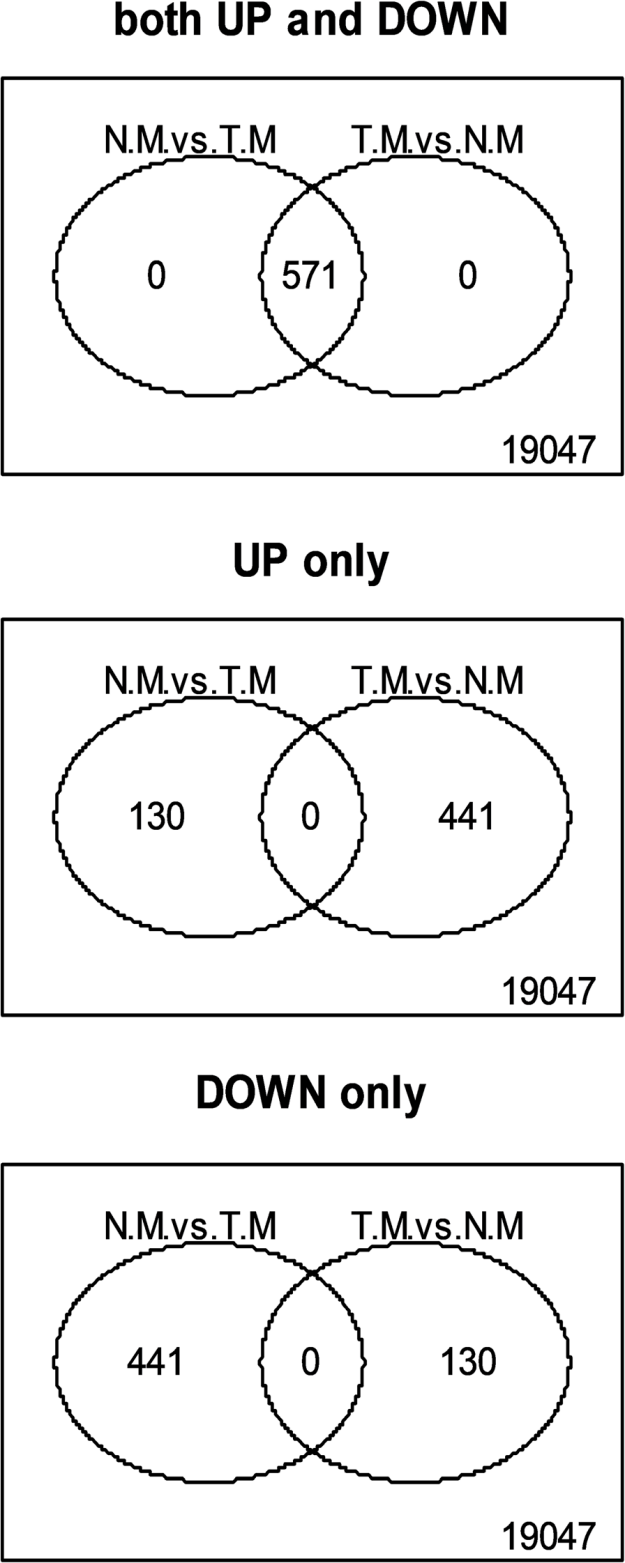
Venn diagrams showing the number of differentially expressed genes (up and down-regulated, only up-regulated or only down-regulated) between normal male (ZH.N.M) and teratological male (ZH.T.M) samples

From a total of 19618 analyzed genes, many were found to be differentially expressed among groups. In total, 2523 genes were differentially expressed between male and female flower buds of a size of 1–6 mm, 733 between male and hermaphrodite flower buds of a size of 1–6 mm and 2165 between hermaphrodite and female flower buds of a size of 1–6 mm (Fig. 1a). Nevertheless, the number of differentially expressed genes increased among flower buds of a size of 7–12 mm. In total, 3144 genes were differentially expressed between male and female flower buds of a size of 7–12 mm, 1427 between male and hermaphrodite flower buds of a size of 7–12 mm and 2884 between hermaphrodite and female flower buds of a size of 7–12 mm (Fig. 1b). Only a total of 571 genes were differentially expressed between normal and teratological male (male to hermaphrodite sex reversal) pistillode (Fig. 2). In general, the number of differentially expressed genes between male and female or hermaphrodite and female flower buds was higher than the number of differentially expressed genes between male and hermaphrodite flower buds.

**Fig. 3:**
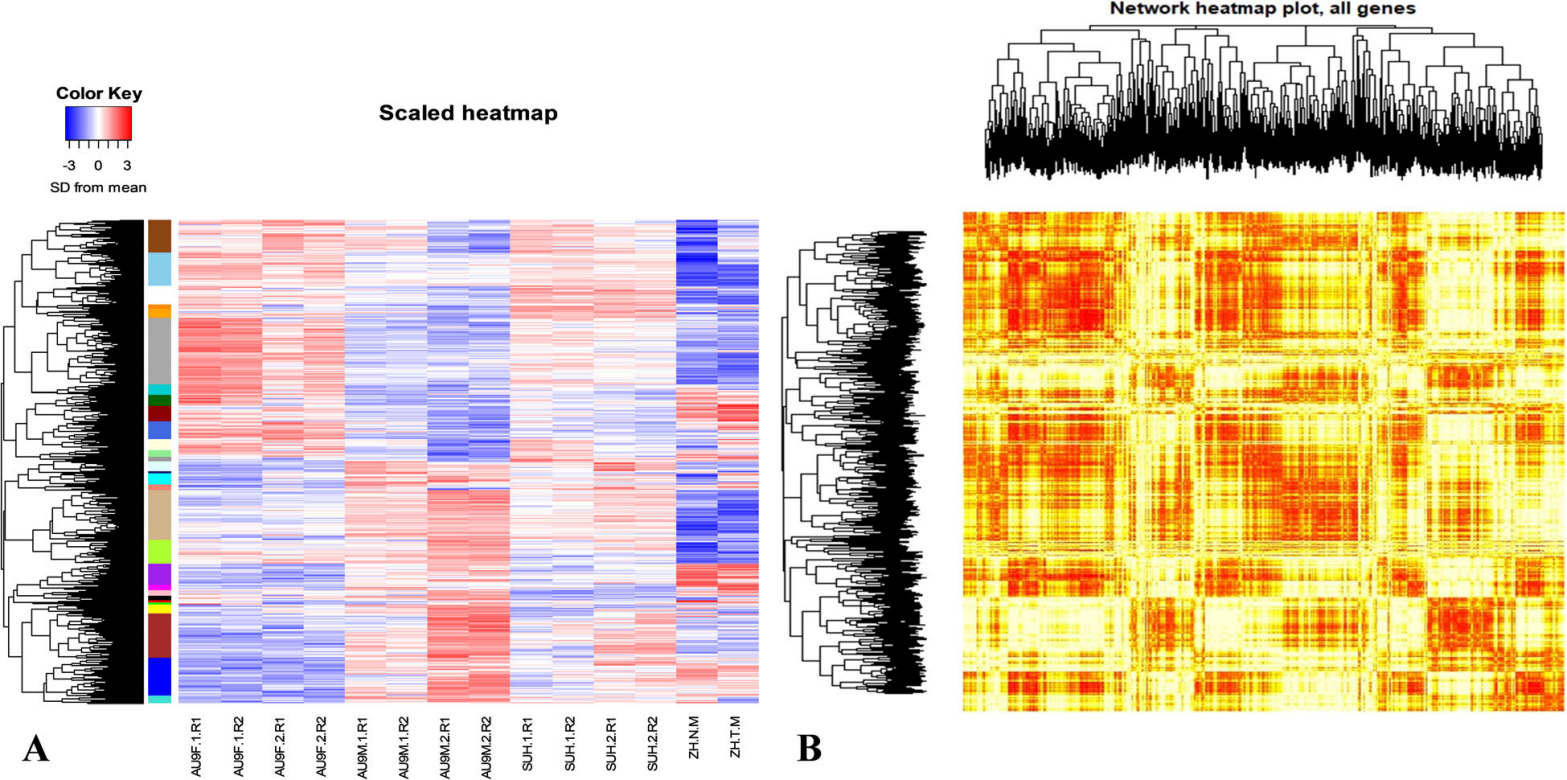
Scaled heatmap (**a**) and TOM plot (**b**) of differential expressed genes (2117 genes) between flower buds of 'AU9' female (AU9F), 'AU9' male (AU9 M) and 'SunUp' hermaphrodite (SUH) with different sizes (1: 1 to 6 mm or 2: 7 to 12 mm) and two replicates (R1: biological replicate 1 or R2: biological replicate 2)

Since the objectives of this study were to identify candidate genes for correct sex expression between males, females and hermaphrodites, and to contribute with the understanding of flower development regulation in papaya among different sex types, only differentially expressed genes between male, female and hermaphrodite flower buds and differentially expressed between normal male and teratological male samples were selected for further analysis (2117 genes in total). A scaled heatmap was built to compare the expression of these genes among the different samples (Fig. 3a). In the heatmap, genes that are up-regulated are shown in red, while genes that are downregulated are shown in blue. The color pattern revealed contrasting expression among samples from different sex, but less contrasting expression among samples from different stages but same-sex (Fig. 3a). Based on these colors, there is a contrast between female and male samples, in which two big groups of genes seem to be overexpressed in females but downregulated in males or overexpressed in males but downregulated in females. This clear pattern is not evident in hermaphrodite samples. In hermaphrodite samples, half of the genes upregulated in females but downregulated in males seemed upregulated, while the other half seemed downregulated and the same seemed to be the case of the genes that are upregulated in males but downregulated in females. The heatmap also reveals a small number of genes showing contrasting expression between teratological and normal male pistillode samples. A TOM (Topological Overlap Matrix) plot was also built to find out the level of complexity of the gene network involved in papaya flower development (Fig. 3b). In this plot, genes that have a similar expression pattern are shown in red, while genes that have no similar expression pattern are shown in yellow (Fig. 3b). The color pattern shown in this figure revealed many clusters of genes or modules that might be part of a similar pathway and a high level of complexity of the gene network for flower development.

### Gene Ontology analysis and over-representation results

**Fig. 4:**
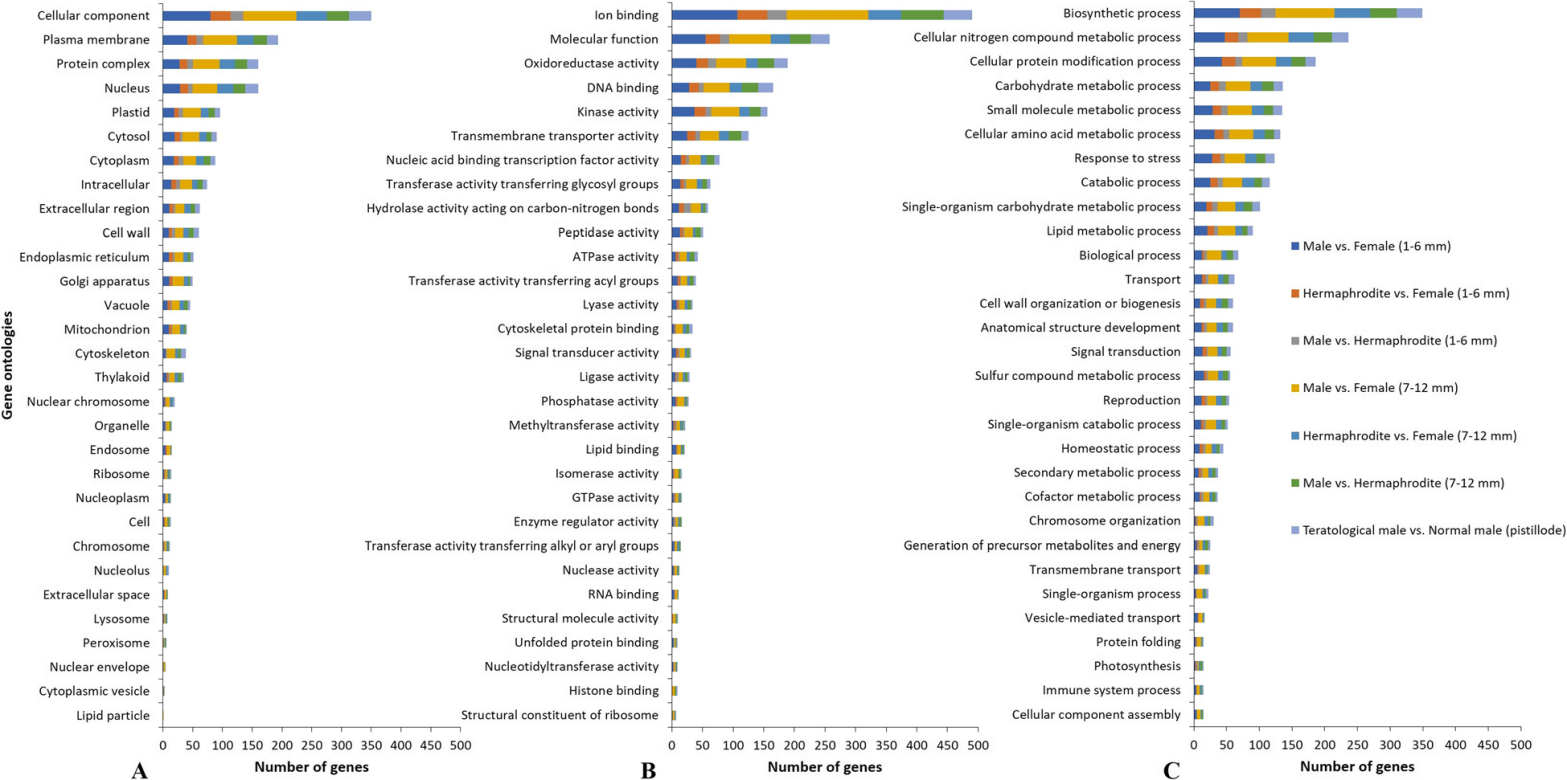
Distribution of annotations for cellular components (**a**), molecular functions (**b**) and biological processes (**c**) for 2117 differentially expressed genes among male, female and hermaphrodite flower buds and between normal male and teratological male samples. Different colors represent the percentage of genes found differentially expressed in each annotation category when doing comparisons among specific samples. Dark blue: Male vs. Female (size: 1–6 mm), Orange: Hermaphrodite vs. Female (size: 1–6 mm), Grey: Male vs. Hermaphrodite (size: 1–6 mm), Yellow: Male vs. Female (size: 7–12 mm), Blue: Hermaphrodite vs. Female (size: 7–12 mm), Green: Male vs. Hermaphrodite (size: 7–12 mm) and Light Blue: Teratological male vs. Normal Male (pistillode)

Gene Ontology annotations for the 2117 selected genes were analyzed and the sequences were classified into three categories according to their GO term: molecular functions (MF), biological process (BP) or cellular components (CC). In total 2081 sequences were classified in the MF category, 2632 in the BP category and 1736 in the CC category (Fig. 4). The most abundant terms for cellular components were plasma membrane, protein complexes, and nucleus (Fig. 4a). The most abundant molecular function terms were for ion binding activity, oxidoreductase activity, DNA binding, kinase activity and transmembrane transporter activity (Fig. 4b). The most abundant biological process terms were for biosynthetic processes, nitrogen metabolism, protein modification, carbohydrate metabolism, amino acid metabolism, response to stress, catabolic processes and single organism carbohydrate processes (Fig. 4c). Figure 4a, b and c also show the percentage of differentially expressed genes found for each annotation category from all individual comparisons made among the sample groups (comparisons are indicated in the figure legend).

Among biological process terms: developmental processes, reproduction and embryo development gene annotations were found (Fig. 4c). Within this last category, genes related to flower development processes and floral organ identity were found as differentially expressed (Tables 1, 2 and 3) and will be further discussed. None of the genes mapped to the available papaya sex chromosome sequences (X, Y or Y^h^), which means that the genes found in this study as differentially expressed among sex-types are not ultimately responsible for sex determination in papaya, but instead might contribute to correct sex expression or development of sex flower organs. Interestingly, the gene which showed the highest fold change between male, hermaphrodite and female flower buds was ‘evm.model.supercontig_2.119’ identified as a PHD-type plant homeodomain protein (PHD finger protein MALE STERILITY 1) (Tables 1 and 2).

**Table 1:** Genes annotated for developmental processes, reproduction and/or embryo development between female, male and hermaphrodite flower buds (size 1 to 6 mm)

Gene ID	Description	LogCPM	Male vs. Female		Male vs. Hermaphrodite		Hermaphrodite vs. Female	
			FC*	FDR*	FC*	FDR*	FC*	FDR*
evm.model.supercontig_2.119	PHD Finger MALE STERILITY 1	5.87	2835.03	2.99E-02	294.20	1.01E-01	9.64	9.69E-01
evm.model.supercontig_157.32	Glucan endo-1,3-beta-glucosidase-like	5.20	30.13	4.50E-02	19.58	1.69E-01	1.54	9.79E-01
evm.model.supercontig_87.13	PHD Finger MALE MEIOCYTE DEATH 1	0.11	21.62	2.82E-02	1.32	1.00E+ 00	16.44	6.83E-02
evm.model.supercontig_85.48	Probable kinase	1.80	9.14	1.72E-02	1.92	1.00E+ 00	4.77	1.68E-01
evm.model.supercontig_50.85	Glycerol-3-phosphate acyltransferase 1	5.70	6.16	4.05E-02	4.31	2.07E-01	1.43	8.91E-01
evm.model.supercontig_1525.1	Abscisic acid 8-hydroxylase 1	5.22	5.32	1.34E-04	3.72	2.57E-03	1.43	3.48E-01
evm.model.supercontig_6.104	Steroid 22-alpha-hydroxylase/ cytochrome P450 90B1-like	3.97	4.73	2.98E-04	1.24	1.00E+ 00	3.81	3.18E-03
evm.model.supercontig_75.17	E3 ubiquitin-protein ligase XBAT32/33	4.14	3.49	1.02E-03	2.23	1.49E-01	1.56	5.40E-01
evm.model.supercontig_26.241	Cytochrome P450 83B1 (CYP83B1)	4.92	2.54	2.93E-02	1.12	1.00E+ 00	2.26	6.18E-02
evm.model.supercontig_427.3	Protein of unknown function (DUF642)	6.77	2.40	1.64E-04	−1.41	3.79E-01	3.38	2.66E-05
evm.model.supercontig_27.166	G-type lectin S-receptor-like serine threonine-kinase RKS1	4.48	2.09	4.30E-02	1.72	4.07E-01	1.21	8.95E-01
evm.model.supercontig_6.12	Pentatricopeptide repeat-containing protein chloroplastic	5.38	2.02	3.09E-03	1.57	1.58E-01	1.29	5.27E-01
evm.model.supercontig_26.316	Floral homeotic protein PISTILLATA	6.99	1.83	4.97E-03	−1.17	1.00E+ 00	2.14	1.44E-03
evm.model.supercontig_16.181	Condensin-2 complex subunit D3 (NCAPD3)	4.54	1.44	5.41E-01	−1.76	3.23E-01	2.53	5.09E-03
evm.model.supercontig_44.60	Transcription factor MYB4-like	1.68	1.16	9.91E-01	8.43	6.06E-02	−7.25	4.32E-02
evm.model.supercontig_59.35	Ammonium transporter 1 member 2	7.05	−1.42	1.95E-01	1.45	3.10E-01	−2.06	7.87E-04
evm.model.supercontig_13.86	Tetraspanin-11 related	2.43	−1.95	6.84E-01	−7.74	1.92E-02	3.96	1.02E-01
evm.model.supercontig_12.194	Scarecrow-like transcription factor PAT1	7.00	−2.09	2.49E-04	−1.62	8.51E-02	−1.29	4.66E-01
evm.model.supercontig_70.45	Potassium transporter 4	7.87	−2.10	2.55E-03	−1.21	1.00E+ 00	−1.73	1.72E-02
evm.model.supercontig_33.178	Calmodulin	9.88	−2.12	7.14E-04	−1.97	5.38E-03	−1.07	9.75E-01
evm.model.supercontig_8.181	Sterol 3-beta-glucosyltransferase UGT80A2	7.17	−2.14	6.60E-04	−1.50	2.37E-01	−1.43	1.93E-01
evm.model.supercontig_18.43	bZIP transcription factor 53	6.73	−2.15	1.32E-04	−1.54	1.42E-01	−1.40	2.19E-01
evm.model.supercontig_27.172	G-type lectin S-receptor-like serine threonine- kinase B120	4.56	−2.20	1.84E-02	−1.21	1.00E+ 00	− 1.82	1.27E-01
evm.model.supercontig_52.111	VAN3-binding/Auxin canalisation (Auxin canalis)	5.61	−2.22	5.19E-04	−1.64	6.37E-02	−1.35	2.98E-01
evm.model.supercontig_132.29	E3 ubiquitin- ligase COP1-like/ Protein suppressor of PHYA-105 1	4.30	−2.22	3.88E-02	1.09	1.00E+ 00	−2.43	2.14E-02
evm.model.supercontig_6.349	Thioredoxin reductase 2-like	5.96	−2.50	8.72E-05	−1.67	3.59E-02	−1.50	7.78E-02
evm.model.supercontig_18.81	Transcription factor UPBEAT1	2.86	−3.24	1.12E-01	−5.37	2.85E-02	1.66	7.36E-01
evm.model.supercontig_25.116	AP2-like ethylene-responsive transcription factor AIL1	2.96	−3.71	4.92E-02	−1.19	1.00E+ 00	− 3.11	1.24E-01
evm.model.supercontig_160.4	Receptor kinase HAIKU2	9.55	−3.71	9.38E-04	−1.95	1.01E-01	−1.90	5.32E-02
evm.model.supercontig_49.19	Abscisic acid 8 -hydroxylase 4	3.51	−4.53	5.74E-03	−6.17	7.33E-03	1.36	8.60E-01
evm.model.supercontig_444.5	Beta-glucosidase 27-like	1.15	−10.02	2.50E-02	−5.43	3.00E-01	−1.85	8.20E-01
evm.model.supercontig_189.36	Beta-glucosidase 32-like	2.34	−17.19	7.54E-03	−3.89	3.65E-01	−4.42	1.15E-01
evm.model.supercontig_1.155	Cytochrome P450 85A-like/ Brassinosteroid-6-oxidase 2 (CYP85A2, BR6OX2)	−0.77	−60.77	3.13E-02	−8.79	7.56E-01	−6.91	2.89E-01

^a^Differentially expressed genes parameters: Fold-Change (FC) > 2 or < − 2 and a False Discovery Rate (FDR) < 0.05

**Table 2:** Genes annotated for developmental processes, reproduction and/or embryo development between female, male and hermaphrodite flower buds (size 7 to 12 mm)

Gene ID	Description	LogCPM	Male vs. Female		Male vs. Hermaphrodite		Hermaphrodite vs. Female	
			FC*	FDR*	FC*	FDR*	FC*	FDR*
evm.model.supercontig_2.119	PHD Finger MALE STERILITY 1	5.87	3350.22	7.72E-03	1.07	1.00E+ 00	3118.56	8.87E-03
evm.model.supercontig_157.32	Glucan endo-1,3-beta-glucosidase-like	5.20	75.59	1.38E-02	2.31	7.94E-01	32.78	3.33E-02
evm.model.supercontig_72.35	Egg cell secreted protein-like	−0.25	56.37	2.66E-02	3.13	6.71E-01	18.04	1.04E-01
evm.model.supercontig_3.299	Cellulose synthase–like protein D1	−0.33	23.88	4.03E-02	1.33	1.00E+ 00	17.98	6.28E-02
evm.model.supercontig_182.26	Cytokinin dehydrogenase 6	0.95	17.90	8.92E-03	8.14	9.47E-02	2.20	7.73E-01
evm.model.supercontig_14.52	Actin cross-linking (DUF569)	0.94	13.07	1.28E-02	6.39	1.35E-01	2.05	7.61E-01
evm.model.supercontig_50.85	Glycerol-3-phosphate acyltransferase 1	5.70	10.39	1.30E-02	−1.20	1.00E+ 00	12.49	1.04E-02
evm.model.supercontig_1525.1	Abscisic acid 8-hydroxylase 1	5.22	9.79	2.90E-05	−1.67	1.26E-01	16.31	1.38E-05
evm.model.supercontig_85.48	Probable kinase	1.80	8.78	1.07E-02	1.21	1.00E+ 00	7.24	2.39E-02
evm.model.supercontig_11.32	Calmodulin-binding receptor-like cytoplasmic kinase 2	4.72	4.03	2.88E-05	1.22	9.77E-01	3.31	8.43E-05
evm.model.supercontig_6.104	Steroid 22-alpha-hydroxylase /cytochrome P450 90B1-like	3.97	3.31	5.19E-03	1.19	1.00E+ 00	2.78	2.40E-02
evm.model.supercontig_27.166	G-type lectin S-receptor-like serine threonine-kinase RKS1	4.48	3.22	2.73E-04	1.88	1.41E-01	1.71	1.98E-01
evm.model.supercontig_89.64	Homeobox-leucine zipper HAT4-like	5.04	3.20	1.94E-05	1.67	1.14E-01	1.92	1.64E-02
evm.model.supercontig_75.17	E3 ubiquitin-protein ligase XBAT32/33	4.14	2.97	4.10E-03	−1.35	9.01E-01	4.02	2.85E-04
evm.model.supercontig_26.316	Floral homeotic protein PISTILLATA	6.99	2.80	1.39E-04	1.30	5.57E-01	2.16	1.03E-03
evm.model.supercontig_26.81	Protein GIGANTEA	7.41	2.51	3.21E-03	1.48	2.67E-01	1.70	6.18E-02
evm.model.supercontig_26.82	GIGANTEA-like	9.07	2.50	1.54E-04	1.40	3.04E-01	1.78	7.36E-03
evm.model.supercontig_23.39	UDP-sugar transporter/Solute carrier family 35 (SLC35D)	4.45	2.38	9.96E-03	1.60	4.23E-01	1.49	4.23E-01
evm.model.supercontig_44.60	Transcription factor MYB4-like	1.68	2.22	5.37E-01	8.95	2.66E-02	−4.03	1.88E-01
evm. TU.contig_29408.2	Metal-nicotianamine transporter YSL3	6.63	2.11	1.14E-04	1.47	1.46E-01	1.43	1.33E-01
evm.model.supercontig_87.13	PHD Finger MALE MEIOCYTE DEATH 1	0.11	1.98	8.37E-01	−8.14	1.53E-01	16.08	2.38E-02
evm.model.supercontig_1.61	Serine threonine-kinase STY46	4.67	1.96	3.69E-02	1.16	1.00E+ 00	1.68	1.54E-01
evm.model.supercontig_20.162	Expansin-like A2	6.35	1.38	1.94E-01	−1.47	1.35E-01	2.03	7.58E-04
evm.model.supercontig_13.86	Tetraspanin-11 related	2.43	−1.01	1.00E+ 00	−7.96	7.60E-03	7.87	4.14E-03
evm. TU.contig_30608.1	CRABS CLAW/ HMG-box domain (HMG box 2)	7.20	−1.50	9.34E-02	1.50	1.80E-01	−2.25	2.14E-03
evm.model.supercontig_97.108	Epidermis-specific secreted glyco EP1-like/ COMITIN	4.66	−1.58	2.26E-01	1.71	2.10E-01	−2.71	8.29E-04
evm.model.supercontig_4.62	Callose synthase 10	4.99	−1.59	3.28E-01	−3.62	1.35E-02	2.28	5.43E-02
evm.model.supercontig_27.172	G-type lectin S-receptor-like serine threonine- kinase B120	4.56	−1.76	1.26E-01	1.21	1.00E+ 00	−2.13	2.44E-02
evm.model.supercontig_16.181	Condensin-2 complex subunit D3 (NCAPD3)	4.54	−1.88	8.63E-02	−2.97	2.11E-03	1.58	2.84E-01
evm.model.supercontig_115.9	Transcription factor HY5	5.54	−1.92	1.10E-03	1.05	1.00E+ 00	−2.01	4.61E-04
evm.model.supercontig_75.60	Protein kinase PINOID 2	5.61	−2.09	1.61E-04	1.25	7.16E-01	−2.61	1.97E-05
evm.model.supercontig_12.194	Scarecrow-like transcription factor PAT1	7.00	−2.13	1.63E-04	−2.10	5.92E-04	−1.01	9.96E-01
evm.model.supercontig_132.29	E3 ubiquitin- ligase COP1-like/ Protein suppressor of PHYA-105 1	4.30	−2.18	4.00E-02	−1.06	1.00E+ 00	−2.06	6.58E-02
evm.model.supercontig_621.3	Major facilitator protein/ Spinster homolog 3	4.27	−2.31	2.98E-02	−1.57	5.77E-01	−1.47	5.17E-01
evm.model.supercontig_84.92	Histone H3 isoform 1	6.36	−2.41	1.58E-04	−1.08	1.00E+ 00	−2.24	3.17E-04
evm.model.supercontig_233.1	AP2-like ethylene-responsive transcription factor AIL5	5.82	−2.54	4.40E-05	−2.28	2.83E-04	−1.11	8.64E-01
evm.model.supercontig_84.93	Histone H3 isoform 2	5.65	−2.85	8.95E-05	1.07	1.00E+ 00	−3.04	7.75E-05
evm.model.supercontig_97.109	COMITIN	2.68	−2.92	2.89E-01	−6.83	2.47E-02	2.34	3.89E-01
evm.model.supercontig_200.13	Cyclin-D3–1	4.91	−3.19	1.38E-04	−2.75	9.81E-04	−1.16	8.45E-01
evm.model.supercontig_3.468	Kinesin-like protein NACK1	4.73	−3.35	1.45E-04	−2.25	1.57E-02	−1.49	3.23E-01
evm.model.supercontig_53.153	Shugoshin-1-like/ Shugosin C terminus	3.12	−3.63	3.33E-02	−3.09	1.28E-01	−1.17	9.58E-01
evm.model.supercontig_81.9	Kinesin family member C1 (KIFC1)	5.45	−3.88	8.46E-05	−1.83	2.73E-02	−2.12	3.39E-03
evm.model.supercontig_125.26	DNA topoisomerase 2 (TOP2)	5.65	−3.93	1.03E-04	−1.80	3.67E-02	−2.18	3.47E-03
evm.model.supercontig_21.170	WUSCHEL-related homeobox 4	4.45	−4.38	1.31E-04	−2.32	3.59E-02	−1.89	9.26E-02
evm.model.supercontig_151.45	1,4-beta-D-xylan synthase	5.73	−4.90	6.66E-05	−2.18	1.27E-02	−2.25	4.04E-03
evm.model.supercontig_160.4	Receptor kinase HAIKU2	9.55	−5.04	2.34E-04	−2.55	1.29E-02	−1.98	3.24E-02
evm.model.supercontig_129.70	AP2-like ethylene-responsive transcription factor ANT	4.92	− 5.60	3.90E-04	−2.22	6.02E-02	−2.52	1.30E-02
evm.model.supercontig_18.81	Transcription factor UPBEAT1	2.86	−5.65	7.56E-03	−4.53	4.53E-02	−1.25	9.29E-01
evm.model.supercontig_160.33	AP2-like ethylene-responsive transcription factor ANT	5.37	−5.94	1.66E-05	−1.42	3.44E-01	−4.19	6.01E-05
evm.model.supercontig_52.111	VAN3-binding/Auxin canalisation (Auxin canalis)	5.61	−6.01	4.68E-06	−1.96	4.19E-03	−3.06	6.53E-05
evm.model.supercontig_25.116	AP2-like ethylene-responsive transcription factor AIL1	2.96	−6.82	1.68E-03	1.67	9.08E-01	−11.43	1.37E-04
evm.model.supercontig_26.241	Cytochrome P450 83B1 (CYP83B1)	4.92	−8.81	4.39E-04	−8.42	1.36E-03	−1.05	9.87E-01
evm.model.supercontig_444.5	Beta-glucosidase 27-like	1.15	−20.46	9.46E-03	−6.73	2.02E-01	−3.04	3.87E-01
evm.model.supercontig_1.155	Cytochrome P450 85A-like/ Brassinosteroid-6-oxidase 2 (CYP85A2, BR6OX2)	−0.77	−104.77	1.73E-02	−8.57	6.54E-01	−12.22	7.15E-02

^a^Differentially expressed genes parameters: Fold-Change (FC) > 2 or < −2 and a False Discovery Rate (FDR) < 0.05

**Table 3:** Genes annotated for developmental processes, reproduction and/or embryo development between normal and teratological male

Gene ID	Description	LogCPM	Normal male vs. Teratological male	
			FC*	FDR*
evm.model.supercontig_6.104	Steroid 22-alpha-hydroxylase /cytochrome P450 90B1-like	3.97	5.91	8.19E-03
evm.model.supercontig_1525.1	Abscisic acid 8-hydroxylase 1	5.22	4.89	1.30E-02
evm.model.supercontig_427.3	Protein of unknown function (DUF642)	6.77	4.33	3.66E-04
evm.model.supercontig_26.316	Floral homeotic protein PISTILLATA	6.99	3.62	1.79E-02
evm.model.supercontig_70.45	Potassium transporter 4	7.87	3.45	4.35E-03
evm. TU.contig_30608.1	CRABS CLAW/ HMG-box domain (HMG box 2)	7.20	3.04	8.59E-03
evm.model.supercontig_7.3	Auxin response factor 3	5.25	2.79	6.33E-03
evm.model.supercontig_2.240	DELLA GAIP-B-like	7.10	−2.16	4.21E-02
evm.model.supercontig_55.116	Floral homeotic protein APETALA 2	6.93	−2.41	1.21E-02
evm.model.supercontig_233.1	AP2-like ethylene-responsive transcription factor AIL5	5.82	−2.63	2.89E-03
evm.model.supercontig_157.48	DNA repair protein RAD50	5.43	−2.76	4.12E-02
evm.model.supercontig_81.9	Kinesin family member C1 (KIFC1)	5.45	−2.83	2.85E-02
evm.model.supercontig_200.13	Cyclin-D3–1	4.91	−2.85	4.18E-02
evm.model.supercontig_52.111	VAN3-binding/Auxin canalisation (Auxin canalis)	5.61	−3.44	2.52E-03
evm.model.supercontig_6.188	3-epi-6-deoxocathasterone 23-monooxygenase (CYP90C1, ROT3)	7.20	−3.90	1.30E-03
evm.model.supercontig_3.468	Kinesin-like protein NACK1	4.73	−4.49	6.47E-03
evm.model.supercontig_129.70	AP2-like ethylene-responsive transcription factor ANT	4.92	−4.92	1.97E-02
evm.model.supercontig_127.38	BTB POZ domain-containing NPY1	5.96	−5.36	1.83E-03
evm.model.supercontig_151.45	1,4-beta-D-xylan synthase	5.73	−5.86	2.76E-03
evm.model.supercontig_125.26	DNA topoisomerase 2 (TOP2)	5.65	−6.34	2.24E-03
evm.model.supercontig_84.93	Histone H3	5.65	−6.46	1.93E-03
evm.model.supercontig_160.33	AP2-like ethylene-responsive transcription factor ANT	5.37	−6.91	6.53E-04
evm.model.supercontig_20.162	Expansin-like A2	6.35	−7.60	1.86E-04
evm.model.supercontig_84.92	Histone H3	6.36	−7.73	4.59E-04
evm.model.supercontig_19.182	cytochrome P450 90A1 (CYP90A1, CPD)	6.70	−8.65	3.44E-05
evm.model.supercontig_97.108	COMITIN	4.66	−16.92	7.80E-04
evm.model.supercontig_21.170	WUSCHEL-related homeobox 4	4.45	−21.19	3.80E-03
evm.model.supercontig_49.19	Abscisic acid 8 -hydroxylase 4	3.51	−21.94	5.76E-03
evm.model.supercontig_25.116	AP2-like ethylene-responsive transcription factor AIL1	2.96	−60.27	7.64E-04

^a^Differentially expressed genes parameters: Fold-Change (FC) > 2 or < −2 and a False Discovery Rate (FDR) < 0.05

Over-represented Gene Ontology (GO) Slim terms (*p*-value < 0,05; FDR < 0,05) were analyzed using the list of differentially expressed genes for each pairwise comparison among sample groups (Additional file 2: Figure S2, Additional file 3: Figure S3 and Additional file 4: Figure S4), to identify differences involved in flower development (common among all sex-types) and important pathways for correct sex expression. As a result, common cellular component terms identified as over-represented were: integral and intrinsic components of membrane; microtubule and microtubule-associated complex; nucleus; polymeric cytoskeletal fiber; supramolecular complex and fiber; and supramolecular complex, fiber and polymer (Additional file 2: Figure S2, shown in blue). Nevertheless, highly over-represented cellular component terms were: chloroplast thylakoid membrane; plant-type vacuole and plastoglobuli (Additional file 2: Figure S2, showed in red). Common molecular function terms identified as over-represented were: transmembrane transporter activity; ATPase activity; catalytic activity; lyase activity; oxidoreductase activity; and transporter activity (Additional file 3: Figure S3, showed in blue). Highly over-represented molecular function terms were: amide transmembrane transporter activity; ATP-dependent microtubule motor activity, peptide, and oligopeptide transmembrane transporter activity (Additional file 3: Figure S3, showed in red). Common biological process terms identified as over-represented were: microtubule-based movement; response to oxygen-containing compounds; and small molecule metabolic process (Additonal file 4: Figure S4, showed in blue). Highly over-represented biological process terms were: inorganic anion transmembrane transport; jasmonate mediated signaling pathway; regulation of defense response, response to stimulus, response to stress, signal transduction, heat and wounding (Additional file 4: Figure S4, showed in red). These results suggest that differentially expressed genes that participate in processes related to response to stress conditions, response to oxygen-containing compounds and external stimuli, as well, as molecular functions related to transmembrane transport and oxidoreductase activity might be considered important for flower development and correct sex expression in papaya.

### RT-qPCR expression analysis of *CpMS1*

Since the ‘evm.model.supercontig_2.119’ or *CpMS1* gene presented extremely highest Fold Change (FC) among sex types during early and late flower developmental stages, the expression of genes that are reported to regulate MALE STERILITY 1 expression in model plants was also examined (Table 4), *CpMS1* over-expression was validated by qPCR in male flower buds and other characteristics of this gene were explored.

**Table 4:** Sampling of genes known to regulate the expression of MS1 in *Arabidopsis* and identified ortholog expression in papaya flower buds

Gene ID	Description	Anther development stage	LogCPM	Flower bud size from 1 to 6 mm						Flower bud size from 7 to 12 mm					
				Male vs. Female		Male vs. Hermaphrodite		Hermaphrodite vs. Female		Male vs. Female		Male vs. Hermaphrodite		Hermaphrodite vs. Female	
				FC*	FDR*	FC*	FDR*	FC*	FDR*	FC*	FDR*	FC*	FDR*	FC*	FDR*
evm.model.supercontig_43.78	MADS BOX Protein SEPALLATA 3 (SEP3)	Stamen initiation	7.58	1.39	3.50E-01	−1.35	7.26E-01	1.87	4.22E-02	1.26	5.28E-01	−1.15	9.94E-01	1.45	2.26E-01
evm.model.supercontig_26.309	Leucine-rich receptor-like protein kinase CLAVATA 1 (CLV1)		4.11	1.79	2.88E-01	1.09	1.00E+ 00	1.64	4.48E-01	2.25	5.35E-02	−1.09	1.00E+ 00	2.45	2.94E-02
evm.model.supercontig_26.316	Floral homeotic protein PISTILLATA (PI)		6.99	1.83	4.97E-03	−1.17	1.00E+ 00	2.14	1.44E-03	2.80	1.39E-04	1.30	5.57E-01	2.16	1.03E-03
evm.model.supercontig_6.199	Floral homeotic protein APETALA 3 (AP3) isoform 1		8.01	1.66	2.51E-02	1.04	1.00E+ 00	1.60	5.06E-02	3.95	4.91E-06	1.78	1.36E-02	2.22	1.28E-04
evm.model.supercontig_6.202	Floral homeotic protein APETALA 3 (AP3) isoform 2		7.19	1.22	6.76E-01	−1.19	1.00E+ 00	1.45	1.63E-01	2.23	6.88E-05	1.37	3.54E-01	1.62	2.98E-02
evm.model.supercontig_12.16	Transcription factor Sporocyteless/Nozzle (SPL/NZZ)	Archesporial initiation	2.34	188.33	8.27E-05	1.10	1.00E+ 00	171.97	1.09E-04	26.51	8.87E-04	−3.25	2.58E-01	86.26	1.45E-04
evm.model.supercontig_107.30	Leucine-rich repeat receptor kinase EMS1/EXS-like	Tapetal cell fate specification	5.01	−1.44	4.14E-01	−1.35	9.00E-01	−1.07	9.93E-01	−2.36	2.15E-02	−1.44	5.70E-01	−1.63	1.90E-01
evm.model.supercontig_14.179	Leucine-rich repeat receptor-like Serine/Threonine-protein kinase RPK2		6.43	−2.07	1.61E-03	1.09	1.00E+ 00	−2.25	9.85E-04	−1.92	2.85E-03	−1.02	1.00E+ 00	− 1.87	4.01E-03
evm.model.supercontig_4.154	Protein Tapetum Determinant 1 (TPD1)		2.51	1.25	9.65E-01	1.14	1.00E+ 00	1.10	9.99E-01	−3.35	1.33E-01	−4.00	1.19E-01	1.19	9.63E-01
evm. TU.contig_28309.2	Transcription factor DEFECTIVE IN TAPETAL DEVELOPMENT AND FUNCTION 1 (TDF1)	Tapetal development	7.89	1145.31	7.53E-05	1.55	9.49E-01	736.60	1.19E-04	1449.31	7.86E-05	−2.15	2.99E-01	3114.34	6.33E-05
evm.model.supercontig_871.3	Transcription factor DYSFUNCTIONAL TAPETUM (DYT)		1.45	31.93	5.98E-04	−1.96	1.00E+ 00	62.65	6.06E-05	24.86	2.45E-03	4.12	2.93E-01	6.03	1.93E-01
evm.model.supercontig_20.95	Transcription factor ABORTED MICROSPORES-like (AMS) isoform 1	Microspore maturation	8.44	1408.47	6.82E-05	1.29	1.00E+ 00	1088.99	9.40E-05	312.19	1.36E-04	−3.39	7.89E-02	1057.12	7.73E-05
evm.model.supercontig_20.94	Transcription factor ABORTED MICROSPORES-like (AMS) isoform 2		4.43	102.16	5.66E-04	2.61	2.58E-01	39.18	3.61E-03	7461.29	5.04E-05	1.89	3.61E-01	3941.34	9.67E-05

**Fig. 5:**
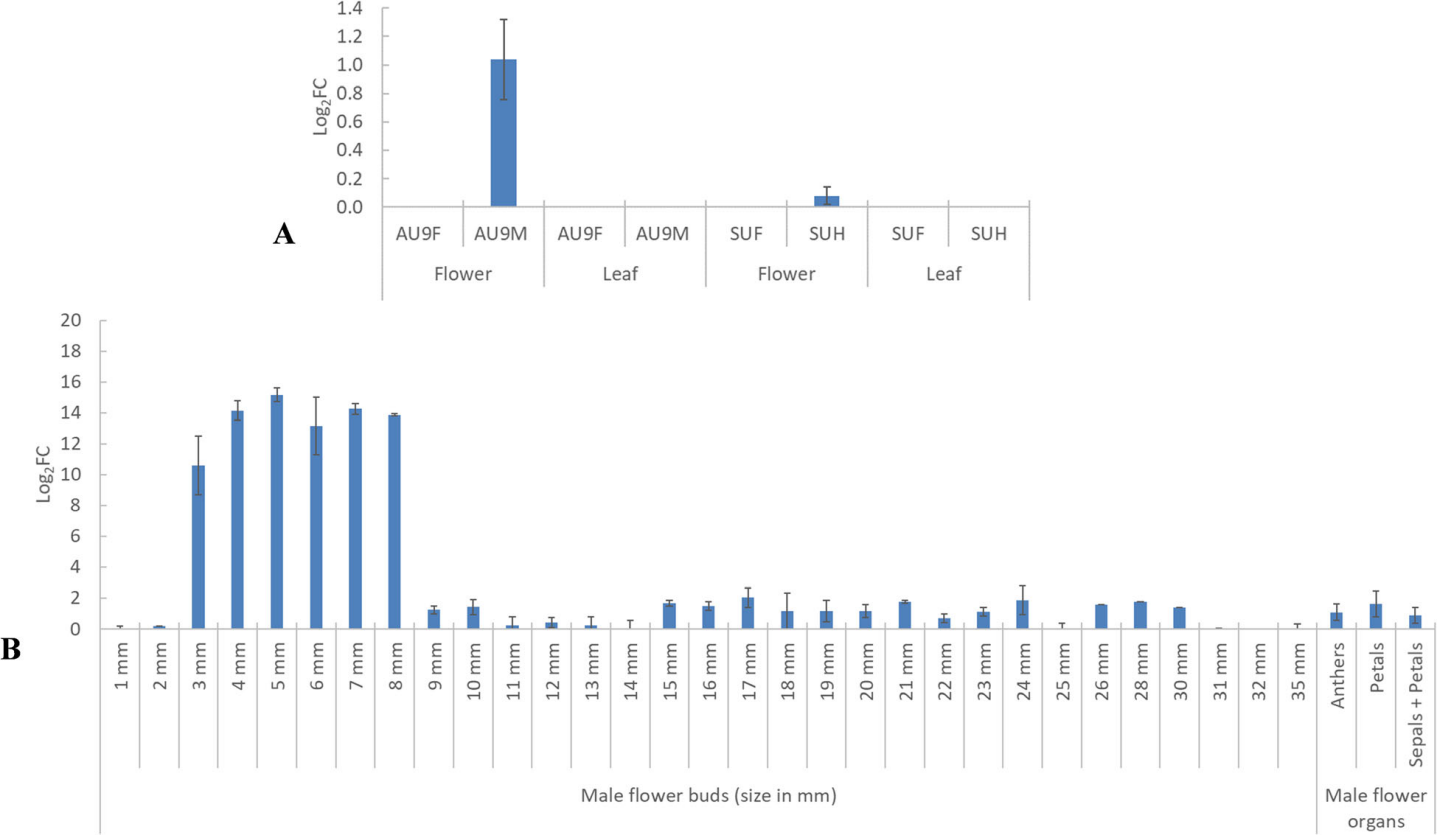
Expression level of *CpMS1* quantified via qRT-PCR in 'AU9' female (AU9F), 'AU9' male (AU9M), 'SunUP' female (SUF) and 'SunUp' hermaphrodite (SUH) flowers compared to leaves (**a**) and on 'AU9' male flower buds of different sizes (mm) and different male flower organs in open male flowers (**b**)

The relative expression or Fold Change (FC) of the PHD finger protein MALE STERILITY 1 was obtained by qPCR and compared among sex-types. Interestingly, this male sterility gene (*CpMS1*) did not amplify in the leaf tissue samples of female, hermaphrodite or male plants; which suggests that its expression is specific for flowers (tissue-specific expression). Furthermore, this gene only amplified in hermaphrodite ‘SunUp’ and male ‘AU9’ flowers, which makes its expression specific for plants with male flower organs, and therefore suggests its participation in male flower organ development in papaya. The evaluation of the expression of *CpMS1* by RT-qPCR showed that it was up-regulated in male flowers in comparison with hermaphrodite flowers (Fig. 5a), which might be explained by a different number of flower buds needed for RNA extraction from hermaphrodite than from male plants, due to the considerable difference in size between hermaphrodite flower buds (larger) and male flower buds (smaller) or even due to differences in the developmental stages of the flower buds that composed each sample. No amplification of the *CpMS1* gene was detected in any of the female flower samples, supporting the RNA-Seq results and *CpMS1* participation on male flower organ development.

Regarding *CpMS1* expression on papaya male flower buds of different size, the gene was significantly up-regulated in flower buds of 3 to 8 mm but was not significantly up-regulated in smaller flower buds (1 or 2 mm), mature flower buds (from 9 to 35 mm) or flower organs from open male flowers (petals, sepals or anthers) (Fig. 5b). A detailed comparison between male and hermaphrodite flower buds was not possible due to a lack of flower bud material representing all these different developmental stages (1 to 35 mm) from hermaphrodite plants. Regardless of the lack of hermaphrodite flower buds for this analysis, the expression of *CpMS1* was not considered to be significantly different between male and hermaphrodite flower buds according to the previous transcriptome analysis (Tables 1 and 2).

### *CpMS1*: homology analysis and genome location

**Fig. 6:**
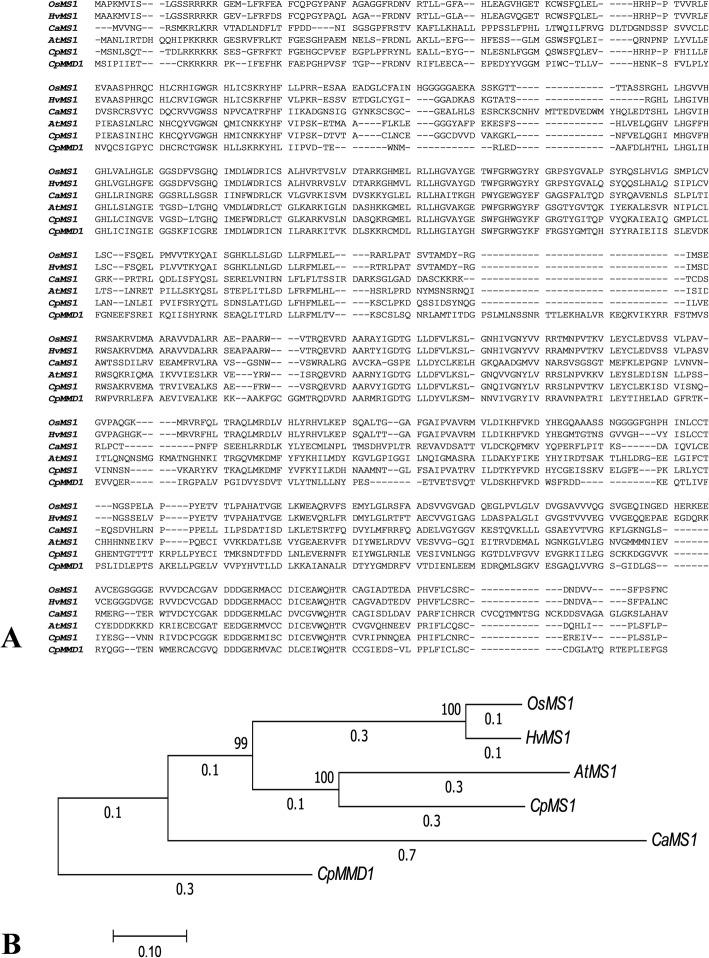
Alignment of MS1 protein sequences from different plant species (**a**) and an evolutionary history tree of *CpMS1* inferred by the Neighbor-Joining method using MEGA7 (**b**)

**Fig. 7:**
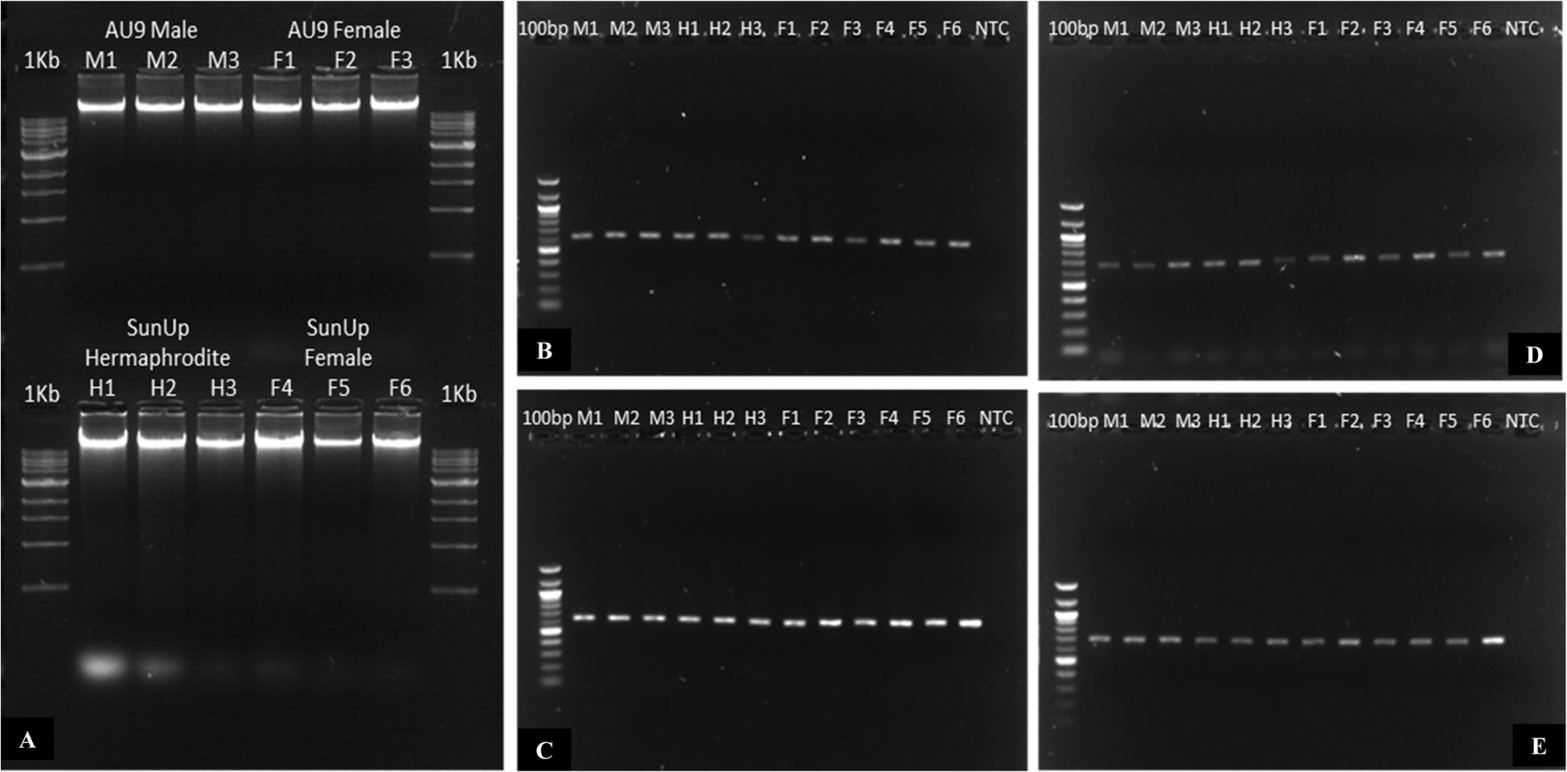
Amplification of *CpMS1* by PCR. **a**. DNA extracted from female, male and hermaphrodite plants. **b**. PCR amplification using primers *CpMS1*–1F and *CpMS1*–1R (up) **c**. PCR amplification using primers *CpMS1*–2F and *Cp MS1*–2R (down). **d**. PCR amplification using primers *CpMS1*–3F and *CpMS1*–3R (up). **e**. PCR amplification using primers *CpMS1*–4F and *CpMS1*–4R (down)

The sequence of the gene identified as PHD finger protein MALE STERILITY 1 (*CpMS1*) in papaya was analyzed and compared to the MALE STERILITY 1 gene found in other species and since its expression was specific for papaya flowers with male organs, its location in the papaya genome was also explored. *CpMS1* contained a unique PHD zinc finger motif (Cys4-His-Cys3), located between the amino acid positions 605 and 653. This protein was highly homologous to other MS1 proteins cloned in other angiosperms plants: *Arabidopsis thaliana* (*AtMS1*) (53.18% identity), *Oryza sativa* (*OsMS1*) (45.17% identity), *Hordeum vulgare* (*HvMS1*) (43.80% identity) and *Capsicum annum* (*CaMS1*) (29.33% identity) (Fig. 6) and which functions have already been well characterized. This gene was located on an autosome (papaya chromosome 02) and no other hit was found for this gene on the papaya genome using cDNA and genomic data. Nevertheless, a single homolog protein was identified in papaya: PHD Finger MALE MEIOCYTE DEATH 1 (‘evm.model.supercontig_87.13’) or *CpMMD1* (Fig. 6), which was also differentially expressed between male and female flower buds of a size 1–6 mm and hermaphrodite and female flower buds of a size 7–12 mm (Tables 1 and 2) according to the previous transcriptome analysis. However, *CpMMD1* did not group with the rest of the MS1 proteins, which indicates that it might have a different function than the one from *CpMS1* (Fig. 6). Unfortunately, the *CpMS1* gene was not classified as a candidate for sex determination, because it amplified using the genomic DNA from the three different sex-types which means that this gene is not located on the Y chromosome (Fig. 7), although its expression was sex-biased (specific to male and hermaphrodite flowers), and its genomic sequence was not different among sex-types.

### Co-expression network of anther development pathway genes

A co-expression correlation network was build using all differentially expressed genes and a sub-network was extracted from this network (Additional file 5: Figure S5) using the *CpMS1* gene, the genes identified as orthologs of genes known to regulate the expression of MS1 in *Arabidopsis thaliana* (Table 4) and their first closest neighbors in the total gene network. This correlation subnetwork had 287 nodes and 4127 edges and included 4 clusters of correlated genes (Additional file 5: Figure S5). The first cluster was the biggest, it included 209 nodes and 3462 edges. This cluster also included the *CpMS1* gene, as well as orthologs of the transcription factors: Sporocyteless/Nozzle (SPL/NZZ), DEFECTIVE IN TAPETAL DEVELOPMENT AND FUNCTION 1 and ABORTED MICROSPORES. The second cluster included the orthologs of the transcription factors: PISTILLATA (PI) and APETALA 3 (AP3), with a positive correlation between them. The third cluster included the protein CLAVATA 1 (CLV1) and the fourth cluster included the transcription factor DYSFUNCTIONAL TAPETUM (DYT). By analyzing the over-representation of biological process annotations of all the genes found in this sub-network (Additional file 6: Figure S6), the following categories with the highest overrepresentation were found: cellular component assembly involved in morphogenesis, pollen development, pollen wall assembly, external encapsulating structure organization, pollen exine formation and sporopollenin biosynthetic processes (Additional file 6: Figure S6).

## Discussion

Differentially expressed genes among papaya flower sex types were detected at early and late developmental stages. The number of differentially expressed genes between male and female or hermaphrodite and female flowers were higher than the number of differentially expressed genes between male and hermaphrodite flowers. Male and hermaphrodite plants are genetically alike, and both have similar versions of a Y chromosome; which could explain a similar pattern of gene expression observed in their flowers [6, 8]. Furthermore, a similar pattern of expression during early developmental stages makes sense, because male and hermaphrodite flower development is very similar until anthers are developed [9, 10]. Nevertheless, the number of differentially expressed genes practically doubled in the latest developmental stage compared to the early developmental stage between male and hermaphrodite plants, which could potentially explain differences observed among sex types.

### Differential expression in the anther development pathway

The major finding of this study was a Male Sterility 1 gene (*CpMS1*) highly up-regulated in male and hermaphrodite flower buds compared to female flower buds, with tissue (only flower buds) and developmental specific (expressed in male flower buds of 3 to 8 mm) expression. Since the differential expression of this gene has not been reported in papaya flower buds before, we explored its regulation and discussed features of this gene. Papaya PHD finger protein MALE STERILITY 1 (MS1), was homologous to *Arabidopsis*, paprika, rice, and barley MS1 proteins. This gene belongs to the PHD-finger family of transcriptions factors. In plants, the PHD (PlantHomeoDomain) transcription factors family has been described as important for several plant development processes, such as pollen maturation, embryo meristem initiation, root development, germination and control of flowering time. It is still unknown what is the specific function of this transcription factor in papaya flowers or its regulation mechanism, but proteins with a PHD motif act as epigenomic effectors, which means that they recognize and bind to histone modifications (e.g. histone methylation), and as a result they activate or repress genes [29]. Little is known about the functions of this protein in papaya, but it is a well-studied gene in other angiosperm species. In *Arabidopsis*, this gene (*AtMS1*) has been described as a transcription factor that regulates male gametogenesis, critical for anthers, pollen and tapetum development and it expresses briefly in the tapetal cells during microsporogenesis, just before microspore release [30–34]. In *ms1 Arabidopsis* mutant plants, the tapetum does not develop correctly, it degenerates abnormally, and the pollen cell wall development is affected; therefore, plants are described as male-sterile because their pollen is not viable. This phenotype suggests that MS1 may modify the transcription of tapetal genes participating in pollen cell wall development and tapetal Programmed Cell Death (PCD) [34]. Genes regulated by MS1 are thought to be involved in the pollen cell wall and coat formation, but this gene also regulates transcription factors involved in pollen production and sporopollenin biosynthesis, as well as certain enzymes (Cysteine proteases) [33]. Over-expression of this gene in *Arabidopsis* results in plants that show late flowering, flowering stems with an increased number of branches and flowers with distorted organs and reduced fertility [33]. Orthologs of the MS1 gene in *Arabidopsis* have been described in other plant species: barley (*HvMS1*) [35], rice (*OsMS1*) [36] and paprika (CA05g06780) [37], all with a similar function. Therefore, we hypothesize that *CpMS1* could have a similar function in papaya due to its homology with the MS1 genes in the other plant species, but more studies are needed to test this hypothesis.

It is important to mention that in other dioecious plant species, like garden asparagus (*Asparagus officinalis*) and kiwifruit (*Actinidia* spp.), genes related to early anther development and male sterility have been found as specific candidates for sex determination [38–41]. In asparagus, a transcriptome analysis of male flower buds revealed male-biased expression of several genes involved in pollen microspore and tapetum development [40]. Identifying differentially expressed genes exhibiting biased expression in asparagus allowed to identify the earliest points within the anther development pathway that could be influenced by a sex-determination gene. Harkness et al. (2015) showed that in asparagus, microspore maturation genes were up-regulated in male and supermale plants, while down-regulated in females. Later, a MYB-like gene expressed only in asparagus male flower buds, called MALE SPECIFIC EXPRESSION 1 (MSE1), was identified as the sex-determination gene [38]. This gene is homologous to the DEFECTIVE IN TAPETAL DEVELOPMENT AND FUNCTION 1 (TDF1) or MYB35 gene in *Arabidopsis,* and it is located in the asparagus Y chromosome [38, 41]. In kiwifruit, a fasciclin-like gene, called *Friendly Boy* (*FrBy*) has been identified as a sex- determination gene [39]. This gene is strongly expressed in tapetal cells at early anther developmental stages, which is believed to contribute to tapetum degradation after programmed cell death (PCD) and it is also located on the kiwifruit Y chromosome [39]. Despite the male and hermaphrodite biased expression pattern observed for the *CpMS1* gene, this gene was found to be autosomal, not Y specific (present in male or hermaphrodite Y chromosomes), and therefore it cannot be considered as the candidate Y specific gene for male sex determination in papaya.

Instead, we hypothesize that this gene is playing an important role in male flower organ development, like anther, pollen and tapetum development in early stages of flower development and that it is acting downstream of gender specification. The over-representation of biological processes related to anther and pollen development in the co-expression correlation subnetwork supports our hypothesis. In addition, it has been previously reported that in papaya male flowers, pollen starts to develop in the anthers of flower buds of a size of 0.6 cm (6 mm) and tetrads are already found in buds of 0.7 and 0.85 cm (7 to 8.5 mm) [42]. This period overlaps with the expression pattern of the *CpMS1* (3 to 8 mm). Furthermore, pollen development in papaya has been described to progress at the same pace in all types of pollen-producing flowers, consistently with pollen development in other plants [43, 44]; therefore, up-regulation of *CpMS1* in small flower buds might be required for tapetum and pollen development in emerging anthers. Nevertheless, more studies are necessary to determine the exact role that *CpMS1* is playing in papaya male flower organ development, as well as other genes found as correlated with the MS1 expression in the network.

In *Arabidopsis*, male flower organ development has been extensively studied and involves a complex network interaction of transcription factors that are expressed in a spatial/temporal manner [45]. MALE STERILITY 1 (MS1) is just one of the last transcription factors involved in this network and it participates in the later stages of tapetum development and pollen cell wall synthesis [33]. Important transcription factors have been reported to act up-stream of MS1 for anther cell specification, like AGAMOUS (AG), SPOROSYTELESS/NOZZLE (SPL/NZZ), SEPALLATA 3 (SEP3), BARELY ANY MERISTEM 1 (BAM1), BARELY ANY MERISTEM 2 (BAM2) and EXCESS MICROSPOROCYTES1/EXTRA SPOROGENOUS CELLS (EMS1/ EXS) [45]. Of these transcription factors, only a homologous gene to SPL/NZZ (‘evm.model.supercontig_12.16’) was identified as differentially expressed between male and female and male and hermaphrodite papaya flower buds (Table 4). The SPL/NZZ gene in *Arabidopsis* encodes a nuclear protein related to MADS-box transcription factors that are essential to produce most anther cells and to regulate microsporogenesis [46, 47].

Other transcription factors upstream of MS1 participate in tapetal development, like DYSFUNCTIONAL TAPETUM 1 (DYT1), DEFECTIVE IN TAPETAL DEVELOPMENT AND FUNCTION 1 (TDF1), ABORTED MICROSPORES (AMS) and MYB80 [45, 48, 49]. Of these transcription factors, homologous genes to DYT1 (‘evm.model.supercontig_871.3’), TDF1 (‘evm. TU.contig_28309.2’) and two different isoforms of AMS (‘evm.model.supercontig_20.94’ and ‘evm.model.supercontig_20.95’) were identified as differentially expressed between male and female and male and hermaphrodite papaya flower buds (Table 4). In *Arabidopsis*, DYT1 encodes a basic helix-loop-helix (bHLH) transcription factor that acts downstream SPL/NZZ and upstream of TDF1, AMS and MS1 [50, 51]. This transcription factor is essential for tapetal gene regulation during tapetal development and it is reported to interact with other bHLH and MYB transcription factors [50, 52]. In *Arabidopsis*, TDF1 encodes an R2R3 MYB transcription factor required for tapetal development that is regulated directly by DYT1 and act upstream AMS [51]. In *Arabidopsis*, AMS is a bHLH protein that functions downstream DYT and upstream MS1 and it is essential for pollen development and pollen cell wall synthesis [53, 54]. It is worth to mention here that two MYB transcription factors have been identified in two different inversions on the Y chromosome [6, 7], but whether these transcription factors participate in any of the steps for anther development in papaya is still unknown.

Overall, the previous results suggest that *CpMS1* overexpression observed in male and hermaphrodite flower buds is probably the consequence of a complex regulatory cascade, regulated by a Y specific gene acting as a stamen promoting factor, as hypothesized by the theory of sex chromosome evolution in plants. More studies are needed to identify the sex-determination gene in papaya on the sex chromosomes that promote male functions.

### Other genes found as differentially expressed among different papaya sex-types

Among the differentially expressed genes annotated as participating in development, reproduction, and embryo development processes between male and hermaphrodite flowers at early stages, we found ABA-8-hydroxylase 1 (‘evm.model.supercontig_1525.1’), which was overexpressed in male flowers, and ABA-8-hydroxylase 4, which was overexpressed in hermaphrodite flowers (‘evm.model.supercontig_49.19’). Interestingly, the same hydrolases were differentially expressed between normal and teratological male-to-hermaphrodite pistillode, being ABA-8-hydroxylase 1 overexpressed in the normal male and ABA-8-hydroxylase 4 overexpressed in teratological male (male-to-hermaphrodite induced plants). Abscisic acid (ABA) is a well-known phytohormone that is involved in the regulation of several plant developmental processes, including seed dormancy and germination, adaptation to environmental stress conditions, mediation of stomatal closure, senescence and flowering time. In *Arabidopsis*, ABA induces flowering via drought stress response (DE response) by inducing the up-regulation of GIGANTEA (GI), CONSTANS (CO) and FLOWERING LOCUS T (FT) [55] and inhibits flowering by inducing the up-regulation of FLOWERING LOCUS C (FLC) [56, 57]. Interestingly, in male flower buds of a size of 7–12 mm, a GIGANTEA (GI) gene (‘evm.model.supercontig_26.81’) was up-regulated significantly compared to female flower buds, while in hermaphrodite flower buds of a size of 7–12 mm, a GIGANTEA-like gene (‘evm.model.supercontig_26.82’) was up-regulated significantly compared to female flower buds.

Among other differentially expressed genes between male and hermaphrodite flowers at later stages, we found several transcription factors. A transcription factor annotated as UPBEAT 1 (‘evm.model.supercontig_18.81’), was overexpressed in hermaphrodite flowers compared to male flowers at early stages. This transcription factor belongs to the bHLH transcription factor family and has been described to regulate the expression of peroxidases that indirectly determine the concentration of reactive oxygen species (ROS) for the differentiation or proliferation of cells at the root meristems in *Arabidopsis* [58, 59]. ROS are known to accumulate in response to stress and are important signaling molecules for the regulation of cell division and differentiation in plants [60]. ROS have been also described to participate in different developmental processes in plants, such as programmed cell death (PCD), seed germination, root growth and root hair development, pollen tube growth and leaf development [61]. In olive (*Olea europaea* L.) hermaphrodite flowers, ROS (H_2_O_2_ and NO) have been reported to accumulate in the reproductive tissues in a developmental dependent manner, with a massive presence on stigmas and anthers, which might be explained by high metabolic activity and cell expansion during the differentiation process [62].

Other transcription factors were overexpressed in hermaphrodite or female flower buds compared to males. Among these transcription factors we found an AP2-like ethylene-responsive transcription factor AIL5 (‘evm.model.supercontig_233.1’) and a WUSCHEL-related homeobox 4 gene (‘evm.model.supercontig_21.170’). AIL5 is an AINTEGUMENTA-LIKE/PLETHORA transcription factor, which is described to play an important role in flower development (especially in floral organ initiation, growth, and patterning), embryogenesis, seedling growth and germination (mediating the repression of gibberellic acid biosynthesis in response to ABA) [63–65]. In *Arabidopsis*, AIL5 is expressed in developing flowers at specific organs (petals, stamens, and carpels) in a similar pattern to AINTEGUMENTA (ANT), and its overexpression produces larger floral organs [63, 66]. Overexpression of AIL5 in hermaphrodite and female flower buds compared to male flower buds makes some sense, because hermaphrodite and female flower buds are bigger than male flower buds and they present bigger flower organs [9, 10, 43]. Interestingly, this transcription factor was also differentially expressed between normal and teratological male-to-hermaphrodite pistillode, being repressed in normal males and overexpressed in teratological males. WUSCHEL-related homeobox 4 (‘evm.model.supercontig_21.170’) was found up-regulated between female and hermaphrodite flower buds compared to male flower buds and up-regulated in teratological male (male-to-hermaphrodite) compared to normal male. WUSCHEL-related homeobox (WOX) proteins are transcription factors that belong to the homeobox protein family on the ZIP superfamily and have a variety of functions in plants, including determining cell fate and lateral organ development [67]. In *Arabidopsis*, 15 WOX genes (including WUSCHEL) have been identified. Some of these WOX genes (including WUSHEL) regulate ovule development, floral organogenesis, floral transition, and participate in gynoecium and embryo development [67, 68]. In *Arabidopsis*, WUSCHEL also activates the AGAMOUS (AG) gene, a class C gene required for normal development of carpels in flowers [69–71]. Other WOX genes in *Arabidopsis* are also capable to alter the expression of the AGAMOUS gene [72].

Here we confirmed the differential expression of important flowering homeotic genes between males or hermaphrodites and females: PISTILLATA (‘evm.model.supercontig_26.316’) and two AP2-like ethylene-responsive transcription factor AINTEGUMENTA (ANT) genes (‘evm.model.supercontig_129.70’ and ‘evm.model.supercontig_160.33’), which were also differentially expressed between males and teratological males (male-to-hermaphrodite). It is well known that PISTILLATA (PI) and AINTEGUMENTA (ANT) are required for proper flower organ development in *Arabidopsis*. PI is required for proper stamen and petal development; while ANT is required for proper flower organ distribution and growth [66, 69, 73–76]. In papaya, the PISTILLATA gene or *Cp*PI has been cloned previously and its expression has been analyzed in male, hermaphrodite and female flower organs. *Cp*PI expression has been reported in petals and stamens of male and hermaphrodite flowers, and only on petals on female flowers [20]. Therefore, this gene was expected to be overexpressed in male and hermaphrodite compared to female flower buds, because female flowers do not present stamens. The down-regulation of *Cp*PI has been reported [16], as well as the up-regulation of two papaya homologous AINTEGUMENTA (ANT) genes, in teratological males (male-to-hermaphrodite) [16], which is consistent with our results. In *Arabidopsis*, besides its role in floral organ growth, ANT participates in the repression of AGAMOUS (AG) expression in the second floral whorl, promotes petal epidermal cell identity and plays an important role on gynoecium and ovule development [77]. Therefore, overexpression of ANT homologous genes in papaya, in female flowers and teratological male (male-to-hermaphrodite) samples compared to males makes sense at early stages of development.

Finally, among differentially expressed genes annotated as participating in development, reproduction, and embryo development processes among male, hermaphrodite and female flowers at early and late stages, we found a VAN3-binding protein. This gene was repressed significantly in male flower buds of 1–6 mm, compared to female flower buds; and in male flower buds of a size 7–12 mm compared to female and hermaphrodite flower buds. In other plants, this protein has been reported to be present in a subpopulation of vesicles from the trans-Golgi-network and to participate in the regulation of the auxin signaling pathway via vesicle transport system [78]. Interestingly, this gene was also differentially expressed in teratological male (male-to-hermaphrodite induced plants) compared to normal male samples. Despite that auxin polar transportation is recognized to play an important role in gynoecium development in *Arabidopsis*, the specific role of this gene in papaya flower development has not been explored [79, 80].

## Conclusions

Our transcriptomic analysis revealed important differences in the expression of genes that participate in developmental, reproduction and embryo development processes among flower buds from plants with different flower sex type. Even though these genes are not located on the sex chromosomes, their differential expression revealed that more studies on anther development, ABA and ROS signaling pathways are required in papaya, to better understand the roles of these genes in flower development or even in sex determination. It is expected that most of these genes act downstream gender specification in papaya and more studies are needed to determine which sex-specific genes on the sex chromosomes are responsible for sex determination. Furthermore, our results confirmed the expression of a gene: *CpMS1* (located on autosomes) in male and hermaphrodite flower buds, which might be required for the normal development of male reproductive organs in papaya. Nevertheless, further studies will be required to elucidate its function and its role in the pathway that regulates male organ development in this species.

## Methods

### Plant material

Flower buds were collected from female and male ‘AU9’ papaya plants and hermaphrodite ‘SunUp’ plants grown at the Kunia Research Station of Hawaii Agriculture Research Center (HARC) in 2013. Papaya ‘AU9’ is a breeding plant material originally from Australia and available at HARC; while papaya ‘SunUp’ is a commercial variety originally from Hawaii available at HARC. The flower buds were used to compare gene expression between sex types and obtain candidate sex-determination genes by RNA-Seq. These flower buds were first classified according to their phenotype (sex) and then were divided into two groups according to their size (in millimeters). One group contained flower buds with a size between 1 and 6 mm (early developmental stages, or pre-meiotic stages) and a second group contained flower buds with a size between 7 and 12 mm (late developmental stages, or post-meiotic stages). Flower buds were ground in liquid nitrogen for further RNA extraction. Two biological replicates were included for each phenotype and for each group. To further corroborate the differential expression of identified highly differentially expressed genes by qPCR, flower buds, and leaf tissue samples were collected again from three different ‘SunUp’ female plants, three different ‘SunUp’ hermaphrodite plants, three different ‘AU9’ female plants and three different ‘AU9’ male plants grown at the Kunia Research Station of HARC during 2017. These samples were collected and used for the qPCR analysis as described below because original flower bud samples from 2013 were not available. All samples were collected in Hawaii by HARC personnel (no required permissions were necessary to collect the samples), shipped in dry ice (−80C) to Urbana, Illinois and then ground in liquid nitrogen (− 196C) for further RNA extraction.

### Total RNA extraction

Total RNA was extracted using 100 mg tissue sample and TRIzol® Reagent (Ambion USA), following the manufacturer’s instructions. After extraction, total RNA was quantified with Nanodrop and its quality was check by electrophoresis (Agarose 1%, TBE 1X Buffer). RNA samples with good quality and quantity were diluted to 100 ng μl^− 1^ and were kept at -80C until further use.

### RNA-Seq library preparation and sequencing

RNA-Seq libraries were constructed using 2 to 2.5 μg of total RNA and the TruSeq® Stranded mRNA LT kit (Illumina USA), following the Low Sample Protocol described by the manufacturer. RNA-Seq libraries were evaluated by electrophoresis (Agarose 1%, TBE 1X Buffer) and quantified with a fluorometer (Qubit® Fluorometer, Invitrogen, USA). RNA-Seq libraries were sequenced using two platforms: HiSeq2000 (single-end, 100 nt) for the first biological replicate and HiSeq2500 (pair-end, 100 nt) for the second biological replicate (Illumina, USA). A summary of the analyzed libraries is presented (Table 5). Besides these libraries, RNA Sequences from normal male (Accession number: SRX1770718) and teratological male (male-to-hermaphrodite sex reversal induced by low temperatures, Accession number: SRX1770817) from a dioecious variety ‘Zhonghuang’, were downloaded from the Sequence Read Archive (SRA) on the National Center for Biotechnology Information (NCBI) database [81] and included in the analysis to identify if genes that were differentially expressed in the “pistillode”, between males and male-to-hermaphrodite sex reversal plants [16]. Raw sequence data for each library is publicly available on Gene Expression Omnibus (GEO, https://www.ncbi.nlm.nih.gov/geo/) under the accession number GSE137547 (BioProject: PRJNA565901, SRA: SRP221947).

**Table 5:** Sample information and details of each library

Sample	Cultivar	Phenotype (sex)	Flower bud size (mm)	Biological replicate	Label	GEO Accession (#)
CP_AU9F_1_R1	'AU9'	Female	1 to 6	1	AU9F.1.R1	GSM4081661
CP_AU9F_1_R2	'AU9'	Female	1 to 6	2	AU9F.1.R2	GSM4081662
CP_AU9F_2_R1	'AU9'	Female	7 to 12	1	AU9F.2.R1	GSM4081663
CP_AU9F_2_R2	'AU9'	Female	7 to 12	2	AU9F.2.R2	GSM4081664
CP_AU9 M_1_R1	'AU9'	Male	1 to 6	1	AU9M.1.R1	GSM4081665
CP_AU9 M_1_R2	'AU9'	Male	1 to 6	2	AU9M.1.R2	GSM4081666
CP_AU9 M_2_R1	'AU9'	Male	7 to 12	1	AU9M.2.R1	GSM4081667
CP_AU9 M_2_R2	'AU9'	Male	7 to 12	2	AU9M.2.R2	GSM4081668
CP_SUH_1_R1	'SunUp'	Hermaphrodite	1 to 6	1	SUH.1.R1	GSM4081669
CP_SUH_1_R2	'SunUp'	Hermaphrodite	1 to 6	2	SUH.1.R2	GSM4081670
CP_SUH_2_R1	'SunUp'	Hermaphrodite	7 to 12	1	SUH.2.R1	GSM4081671
CP_SUH_2_R2	'SunUp'	Hermaphrodite	7 to 12	2	SUH.2.R2	GSM4081672
SRX1770718^a^	'Zhonghuang'	Normal male	NA	1	ZH.N.M	–
SRX1770817^a^	'Zhonghuang'	Teratological male	NA	1	ZH.T.M	–

^a^Source: data generated by [16] and downloaded from the SRA at NCBI

### Differential gene expression analysis

After RNA sequencing, raw read quality was analyzed using FastQC (Version 0.11.5) [82] and adapters and low-quality reads were removed using Trimmomatic (Version 0.36) [83]. Following trimming, raw reads were aligned to the new papaya genome assembly (Papaya PacBio assembly, 280.5 Mb) using Hisat2 (Version 2.0.5) [84]. After alignment, SAM files were converted to BAM files using samtools (Version 1.3.1) [85] and aligned reads were counted using featureCounts (Version 1.5.2) [86]. Reads aligned to exons were counted and summarized per gene ID. Therefore, an annotation file (gff3 files) was generated using GMAP (Version 2013–11–27). The annotation file was generated using papaya coding sequences from Phytozome v.12 (Cpapaya_113_ASGPBv0.4.cds.fa.gz, Version 12-29-2015) and a new papaya genome assembly (Papaya PacBio assembly, 280.5 Mb). The gff3 files were transformed to gtf files using gffread (Version 0.9.8) to count the number of aligned reads, as described above.

Differential gene expression between samples was analyzed using R (Version 3.2.3) and Rstudio (Version 1.0.136) with the following packages edgeR (Version 3.12.1), WGCNA (Version 1.51) and limma (Version 3.26.9). The contrast matrix used for the analysis included all pairwise comparisons between all groups. Only the genes with a Logarithmic Fold Change (Log_2_FC) > 1 or < − 1 (or a Fold Change > 2) and a False Discovery Rate (FDR) < 0.05 were consider as truly differentially expressed. A heatmap was built in R using all identified differentially expressed genes. Gene Ontology (GO) for 2117 selected differentially expressed genes were analyzed with Blast2GO Basic (Version 4.1.9) to reveal GO categories of differentially expressed genes [87–90]. A GO-Slim functional over-representation analysis based on the list of differentially expressed genes in each of the conditions (male vs. female; male vs. hermaphrodite and hermaphrodite vs. female at different sizes 1–6 mm and 7–12 mm) was performed using PANTHER database [91] and the respective gene ID for the corresponding *Arabidopsis* homolog, to reveal differential over-represented GO terms between each of condition. To check whether the 2117 differentially expressed genes belonged to a sex chromosome or to an autosome, genes that were differentially expressed were blasted and mapped to the assembled sex chromosomes pseudomolecules (X, Y, and Y^h^) [6, 7]. No match was found and none of the genes could be mapped back to the sex chromosome pseudomolecules.

### RT-qPCR expression analysis to validate differential expression of *CpMS1*

Total RNA extracted from 100 mg of frozen ground flower buds and leaf tissue samples from wild type ‘SunUp’ female and hermaphrodite plants; and wild type ‘AU9’ female and male plants were treated with DNAse I (ThermoScientific) and 2.0 μg were converted to cDNA with the High Capacity cDNA Reverse Transcription Kit (Applied Biosystems) in a 20 μl reaction, following the steps described by the manufacturer. The relative expression or Fold Change (FC) of the highly differentially expressed gene *CpMS1* (identified by RNA-Seq) was evaluated by qPCR using specific primers (Table 5), 10 ng of cDNA and the PowerUp™ SYBR™ Green Master Mix (Applied Biosystems) in a CFX96™ Real-Time PCR Detection System (BioRad) with a standard cycling mode (Tm 58C) and including a dissociation curve as a final step. Three biological replicates, three experimental replicates and three non-template controls (NTC) were used. Relative gene expression was normalized against three different internal endogenous genes (Actin 2, EIF1 and TBP1) and the respective variety female sample as reference. The ΔΔCt method was used to calculate the relative expression, where Fold Change (FC) for each gene = 2^-(ΔΔCt) and the log Fold Change = Log_2_(FC). Significant differences in Log_2_(FC) were analyzed with an ANOVA and a Tukey test (α = 0.05). The expression of this gene was also evaluated by RT-qPCR in male flower buds classified in different developmental stages by their respective sizes in millimeters (from 1 to 35 mm); and in petals, sepals and anthers from fully developed open male flowers, as described previously. A detailed comparative analysis between male and hermaphrodite flower buds was not possible due to a lack of material representing all the different flower stages (1 mm to 35 mm) from hermaphrodite plants.

### A highly differentially expressed gene *CpMS1*: homology analysis and genome location

Genomic and protein sequences for the highly differentially expressed gene: ‘evm.model.supercontig_2.119’ (*CpMS1*) were extracted from Phytozome (v12.1). Three different databases were used to analyze protein motifs present in the protein sequence: PFAM database [92], SMART database [93] and NCBI Conserved Domains Database [94]. BLASTn was used to analyze the position and the number of copies of the gene in the papaya genome. BLASTp was used to find homologous proteins in the papaya genome. The previous and the new papaya genome assembly (Papaya PacBio assembly, 280.5 Mb) were used to locate and count the number of copies of the gene in the papaya genome. To find out whether this gene was sex-specific or not, primers were designed to amplify the whole gene in segments of 700–800 bp by PCR and DNA from three biological replicates (wild type ‘SunUp’ female and hermaphrodite plants and wild type ‘AU9’ female and male plants) were used. A PCR standard 10 μl reaction composed by *Taq* DNA Polymerase with Standard *Taq* Buffer (NEB), 0.5 ng of DNA and 0.5 μM of the four different specific primer pairs for *CpMS1* (Table 6) were used in a GeneAmp® PCR System 9700 thermal cycler (Applied Biosystems) using the recommended manufacturer thermocycling conditions (Tm 55C). All PCR products were sequenced by Sanger Sequencing in the Roy J. Carver Biotechnology Center at the University of Illinois at Urbana-Champaign, assembled using ChromasPro (version 2.1.8), and compared to the *CpMS1* genomic reference sequence. Orthologs for this gene in other species (*At*MS1, *Hv*MS1, *Os*MS1, and *Ca*MS1), as well as homologs in papaya, were aligned with MUSCLE [95] and compared to the *CpMS1* papaya protein reference sequence using MEGA7 [96].

**Table 6:** Primer pairs for RT-qPCR and PCR of *CpMS1*

Gene	Primer F	Primer R	Tm (C)
Actin 2	TTTCCAAGGGTGAGTATGATGAG	ACACAGGACACAAAAGCCAACTA	58
EIF 1	AGGCAGGCAAGAGAAGAT	TTCATACCGAGTAGCGATTC	58
TBP 1	GGTAGTAGTAGTTAGGTATGTG	GGCAATCTGGTCTCACTT	58
*CpMS1*	ATGGTCAGCTAAGCGAGTTG	CGGGTTTAAGCTACGACGAA	58
*CpMS1*–1	TTGGAAATTAACAAAACGAGAAA	TGGAGAGATTTCTTCAAAAGTTG	55
*CpMS1*–2	GCAATGGCCTCTTTGTTGTT	TCCTGCCTTCCAAAAGATCA	55
*CpMS1*–3	TCAACTCGCTTAGCTGACCA	TGTGAAGGTGGGTGTGATGT	55
*CpMS1*–4	AACCCACTTCGACTCCATTG	TTTCTCCATTTGCAGTGTTTCTT	55

### Co-expression network analysis

A co-expression correlation network was built in CytoScape [97] using the Expression Correlation App, and the expression matrix containing the normalized expression values for all differentially expressed genes. A sub-network was extracted from this co-expression correlation network using the genes identified as the orthologs of genes known to regulate the expression of MS1 in *Arabidopsis thaliana* (Table 4), the *CpMS1* gene and all their first closest neighbors in the co-expression network. To determine which biological process was statistically over-represented in this sub-network, a Hypergeometric test with multiple test correction (Benjamini and Hochberg FDR correction) and a significance level of 0.05 was done in CytoScape using the BiNGO App [98].

## Supplementary information

**Additional file 1: Figure S1.** A cluster of samples based on the normalized LogCPM values (A) and calculated the biological coefficient of variation (B) after RNA-Seq analysis

**Additional file 2: Figure S2.** Fold enrichment of GO-Slim Cellular component terms identified as over-represented.

**Additional file 3: Figure S3.** Fold enrichment of GO-Slim Molecular function terms identified as over-represented.

**Additional file 4: Figure S4.** Fold enrichment of GO-Slim Biological process terms identified as over-represented.

**Additional file 5: Figure S5.** Co-expression correlation sub-network of genes in the anther developmental pathway.

**Additional file 6: Figure S6.** Over-represented biological processes in the co-expression correlation sub-network.

**Additional file 7: Table S1.** Summary of quality control with FastQC, alignment with HISAT2 and read count with featureCounts.

## Data Availability

The datasets used and/or analyzed during the current study are publicly available on Gene Expression Omnibus (GEO, https://www.ncbi.nlm.nih.gov/geo/) under the accession number GSE137547 (BioProject: PRJNA565901, SRA: SRP221947).

## Abbreviations

ABA: Abscisic Acid
miRNAs: MicroRNAs
RNA: Ribonucleic Acid
RNA-Seq: Ribonucleic acid sequencing
ROS: Reactive Oxygen Species
RT-qPCR: Quantitative Reverse Transcription PCR
SuperSAGE: Improved variant of Serial Analysis of Gene Expression
