# Supplementary figures and images for: Differential gene expression among three sex types reveals a MALE STERILITY 1 (CpMS1) for sex differentiation in papaya
Source: BMC Plant Biol. 2019 Dec 9;19:545. doi: 10.1186/s12870-019-2169-0 (PMC6902354; doi:10.1186/s12870-019-2169-0)

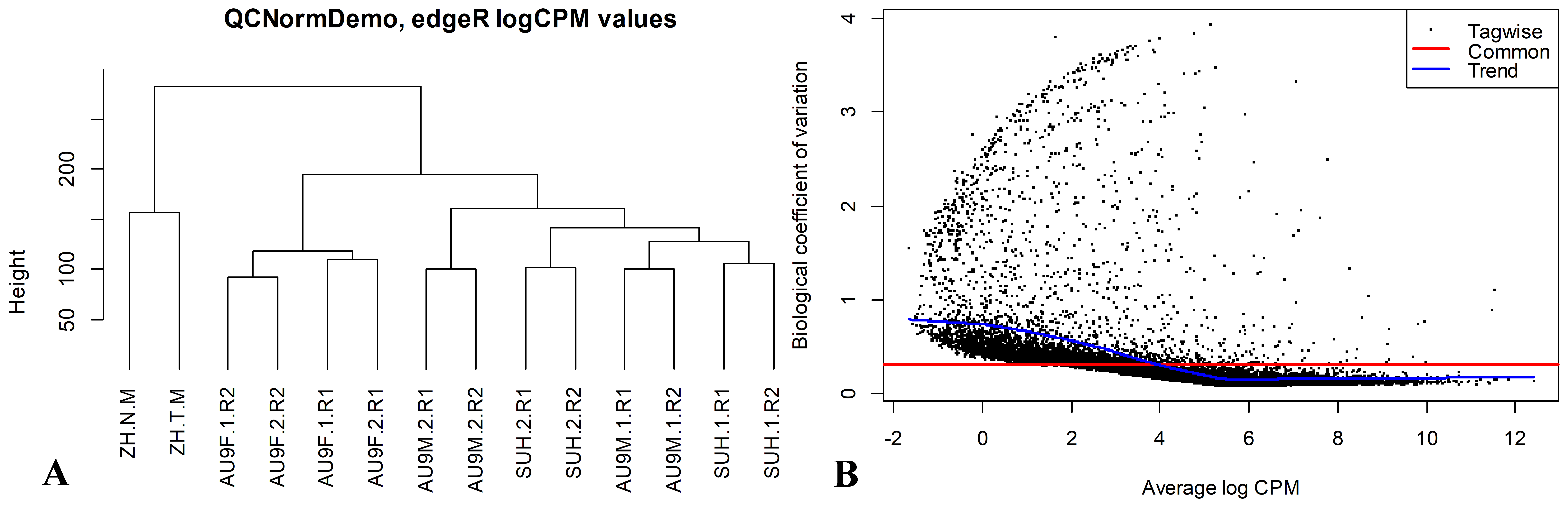

Supplement: Supplementary file 1 — Additional file 1: Figure S1. A cluster of samples based on the normalized LogCPM values (A) and calculated the biological coefficient of variation (B) after RNA-Seq analysis [file 12870_2019_2169_MOESM1_ESM.tif]

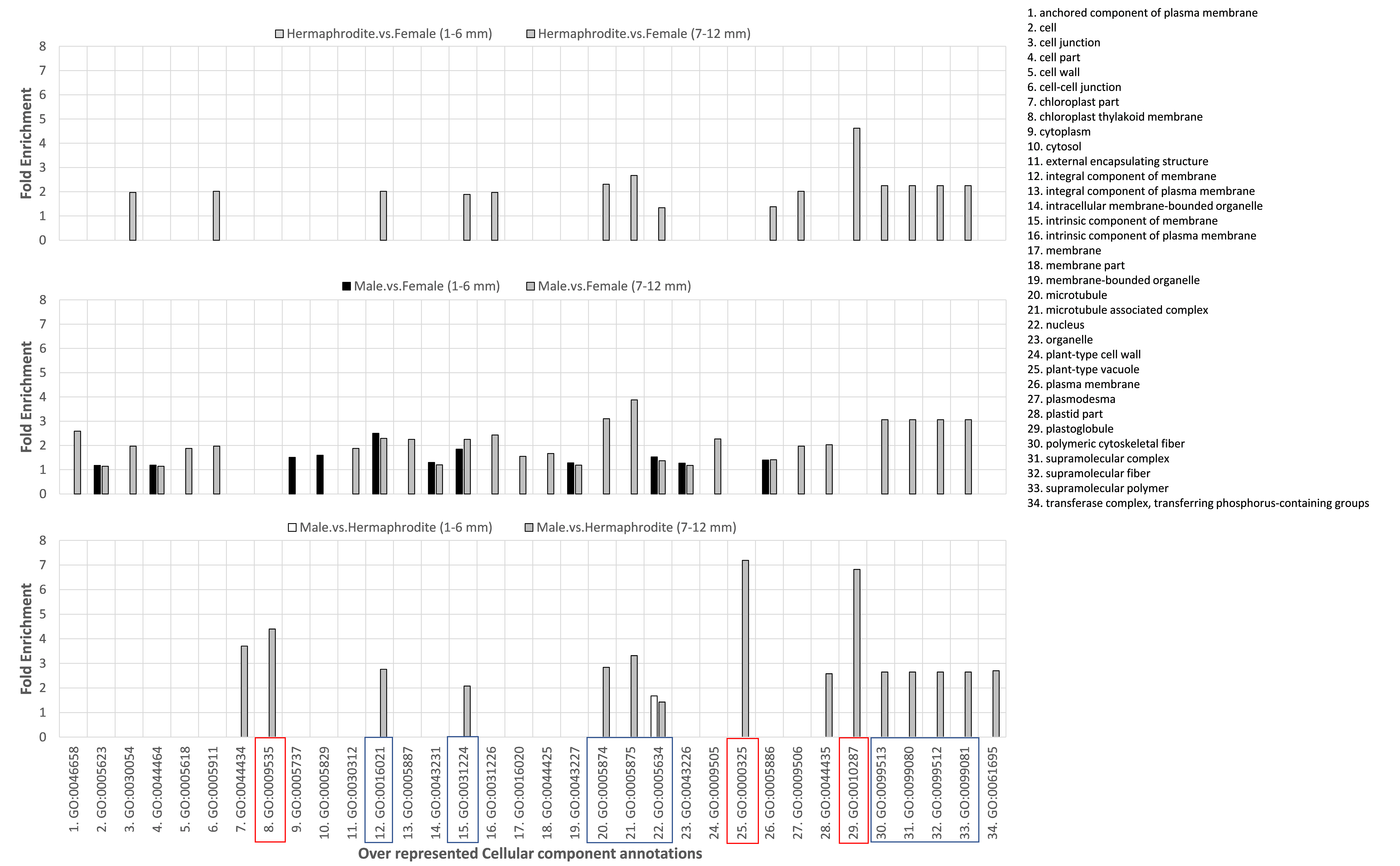

Supplement: Supplementary file 2 — Additional file 2: Figure S2. Fold enrichment of GO-Slim Cellular component terms identified as over-represented. [file 12870_2019_2169_MOESM2_ESM.tif]

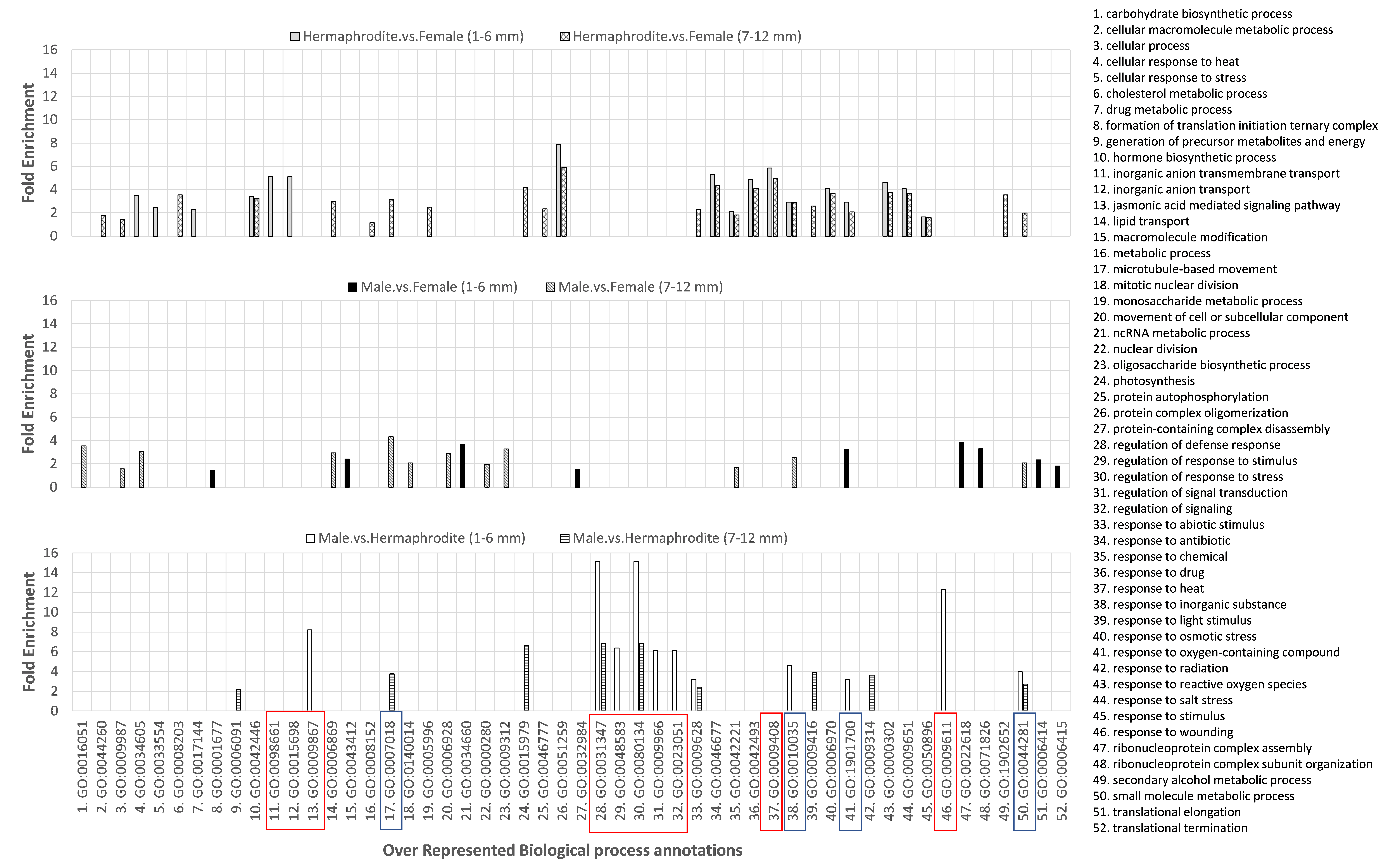

Supplement: Supplementary file 4 — Additional file 4: Figure S4. Fold enrichment of GO-Slim Biological process terms identified as over-represented. [file 12870_2019_2169_MOESM4_ESM.tif]

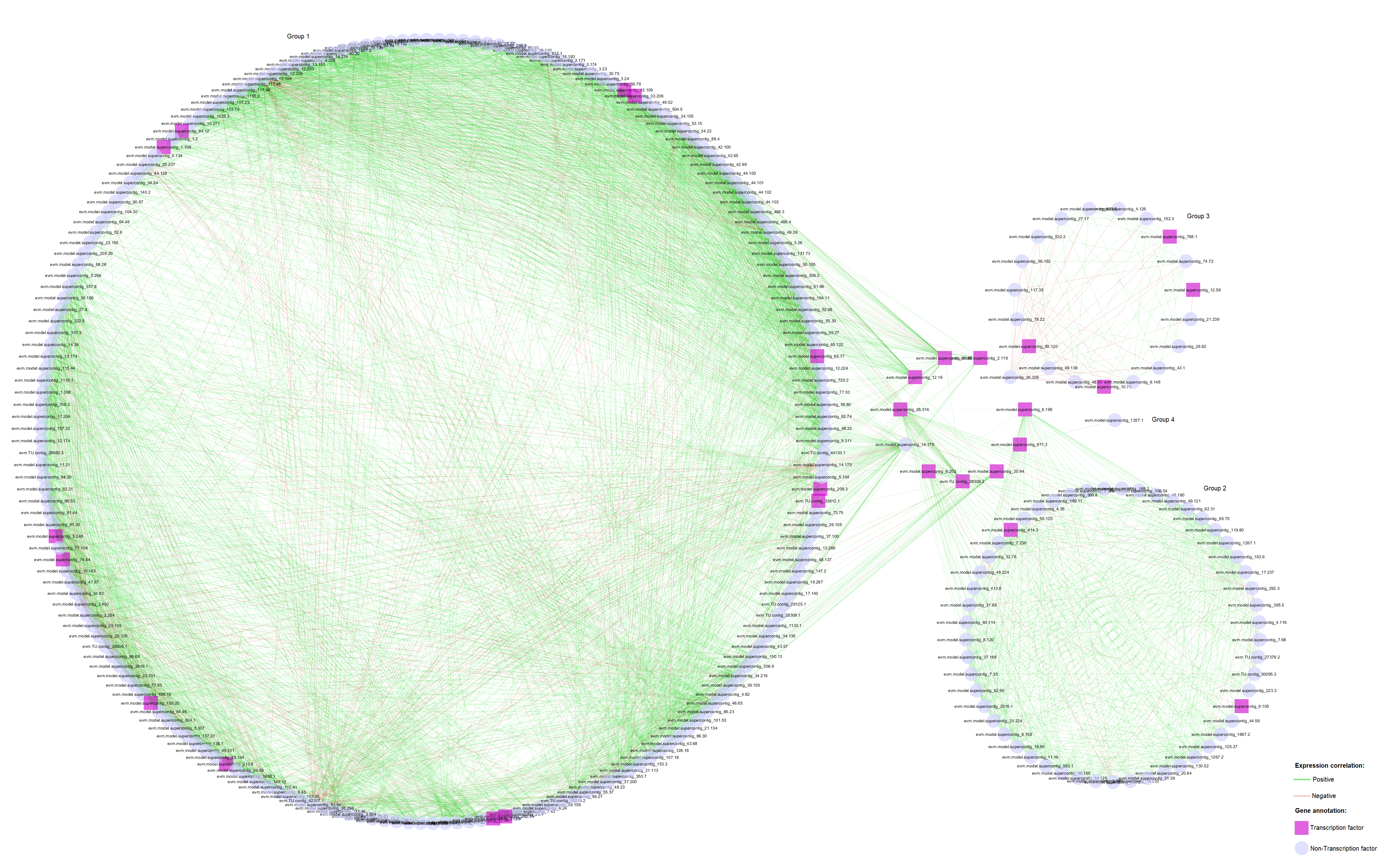

Supplement: Supplementary file 5 — Additional file 5: Figure S5. Co-expression correlation sub-network of genes in the anther developmental pathway. [file 12870_2019_2169_MOESM5_ESM.tif]

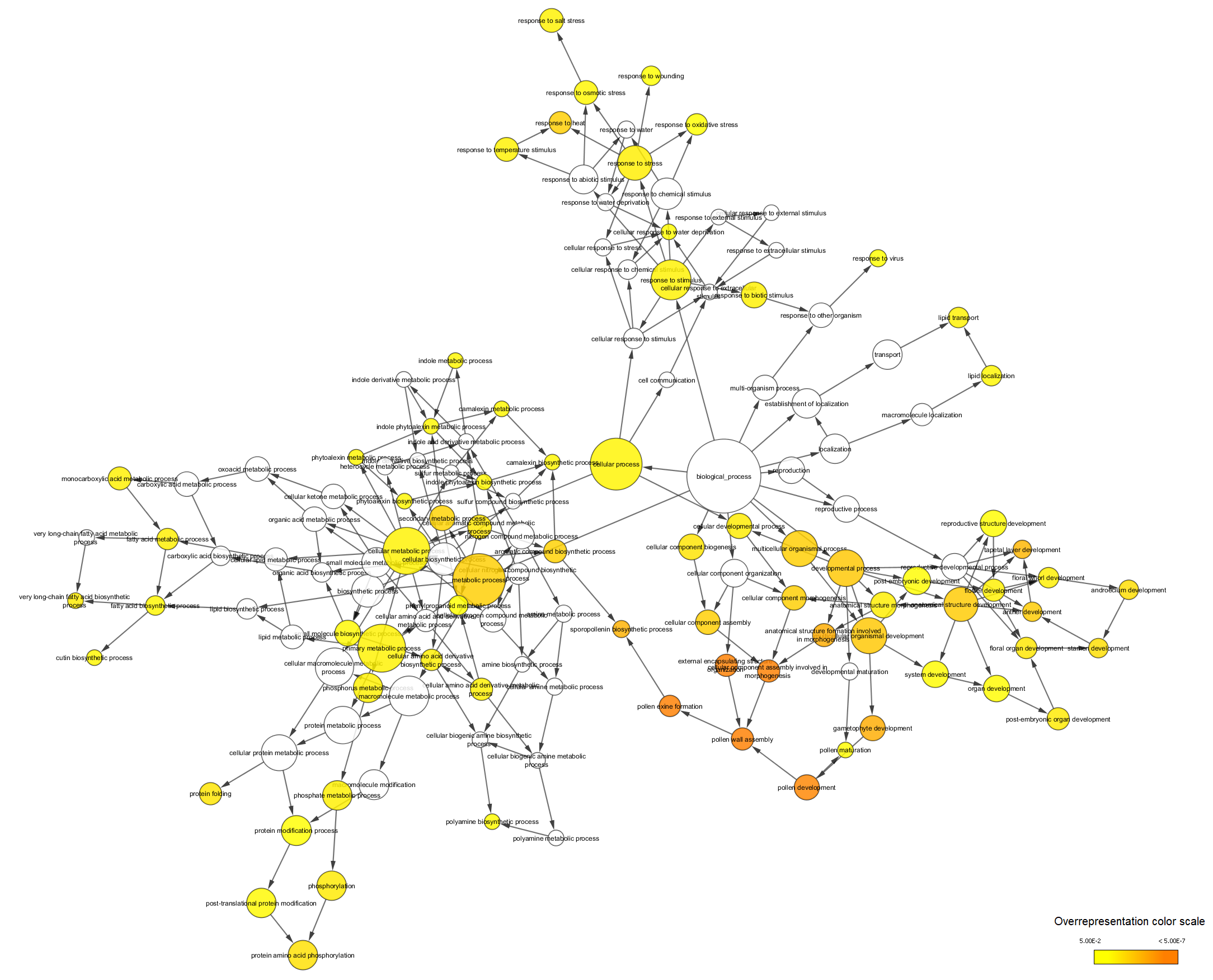

Supplement: Supplementary file 6 — Additional file 6: Figure S6. Over-represented biological processes in the co-expression correlation sub-network. [file 12870_2019_2169_MOESM6_ESM.tif]
