# Supplementary material for: Differential gene expression among three sex types reveals a MALE STERILITY 1 (CpMS1) for sex differentiation in papaya
Source: BMC Plant Biol. 2019 Dec 9;19:545. doi: 10.1186/s12870-019-2169-0 (PMC6902354; doi:10.1186/s12870-019-2169-0)
Supplement: Supplementary file 7 — Additional file 7: Table S1. Summary of quality control with FastQC, alignment with HISAT2 and read count with featureCounts. [file 12870_2019_2169_MOESM7_ESM.docx]

**Additional file 7: Table S1.** Summary of quality control with FastQC, alignment with HISAT2 and read count with featureCounts

| **Sample** | **Description** | **QC data** | | |  | **HISAT2 Summary** | | |  | **featureCounts Summary** | | | | | | |
| --- | --- | --- | --- | --- | --- | --- | --- | --- | --- | --- | --- | --- | --- | --- | --- | --- |
|  |  | **Total**  **Reads**  **(#)** | **Trimmed**  **Reads**  **(%)** | **Survived**  **Reads**  **(%)** |  | **Aligned 1 time**  **(%)** | **Aligned >1 times**  **(%)** | **Non**  **Aligned**  **(%)** |  | **Mapped**  **Genes**  **(#)** | **Mapped**  **Genes**  **(%)** | **Assigned**  **Reads**  **(#)** | **Assigned**  **Reads**  **(%)** | **Unmapped**  **Reads**  **(#)** | **Ambiguous**  **Reads**  **(#)** | **No feature**  **(#)** |
| CP_AU9F_1_R1 | Female, 1-6 mm | 2.25E+07 | 0.07 | 99.93 |  | 87.81 | 7.07 | 5.05 |  | 20279 | 73.24 | 1.52E+07 | 66.15 | 1.13E+06 | 3.63E+05 | 6.28E+06 |
| CP_AU9F_1_R2 | Female, 1-6 mm | 2.56E+07 | 0.20 | 99.80 |  | 86.30 | 7.80 | 5.69 |  | 20931 | 75.59 | 8.55E+06 | 32.75 | 1.46E+06 | 2.08E+05 | 1.59E+07 |
| CP_AU9F_2_R1 | Female, 7-12 mm | 2.15E+07 | 0.06 | 99.94 |  | 87.77 | 7.00 | 5.17 |  | 20193 | 72.93 | 1.45E+07 | 65.98 | 1.11E+06 | 3.59E+05 | 6.03E+06 |
| CP_AU9F_2_R2 | Female, 7-12 mm | 2.26E+07 | 0.22 | 99.78 |  | 86.39 | 7.88 | 5.51 |  | 20956 | 75.68 | 7.43E+06 | 32.08 | 1.25E+06 | 1.98E+05 | 1.43E+07 |
| CP_AU9M_1_R1 | Male, 1-6 mm | 2.45E+07 | 0.06 | 99.94 |  | 87.80 | 7.01 | 5.13 |  | 20718 | 74.82 | 1.66E+07 | 65.97 | 1.26E+06 | 4.26E+05 | 6.86E+06 |
| CP_AU9M_1_R2 | Male, 1-6 mm | 2.25E+07 | 0.24 | 99.76 |  | 75.36 | 7.00 | 17.40 |  | 20989 | 75.80 | 6.59E+06 | 28.58 | 3.92E+06 | 1.78E+05 | 1.24E+07 |
| CP_AU9M_2_R1 | Male, 7-12 mm | 1.98E+07 | 0.06 | 99.94 |  | 81.93 | 7.31 | 10.71 |  | 20577 | 74.31 | 1.27E+07 | 62.51 | 2.12E+06 | 4.36E+05 | 5.07E+06 |
| CP_AU9M_2_R2 | Male, 7-12 mm | 2.17E+07 | 0.19 | 99.81 |  | 82.23 | 8.04 | 9.54 |  | 20920 | 75.55 | 6.77E+06 | 30.43 | 2.07E+06 | 2.22E+05 | 1.32E+07 |
| CP_SUH_1_R1 | Hermaphrodite, 1-6 mm | 2.12E+07 | 0.06 | 99.94 |  | 91.81 | 6.40 | 1.73 |  | 20471 | 73.93 | 1.49E+07 | 68.28 | 3.67E+05 | 3.45E+05 | 6.19E+06 |
| CP_SUH_1_R2 | Hermaphrodite, 1-6 mm | 1.93E+07 | 0.41 | 99.59 |  | 59.52 | 5.42 | 34.65 |  | 20324 | 73.40 | 4.52E+06 | 22.67 | 6.70E+06 | 1.06E+05 | 8.62E+06 |
| CP_SUH_2_R1 | Hermaphrodite, 7-12 mm | 2.32E+07 | 0.06 | 99.94 |  | 91.15 | 6.83 | 1.96 |  | 20597 | 74.39 | 1.63E+07 | 68.48 | 4.54E+05 | 4.04E+05 | 6.66E+06 |
| CP_SUH_2_R2 | Hermaphrodite, 7-12 mm | 2.32E+07 | 0.20 | 99.80 |  | 89.42 | 7.33 | 3.05 |  | 21080 | 76.13 | 8.07E+06 | 33.95 | 7.07E+05 | 1.93E+05 | 1.48E+07 |
| SRX1770718 | Normal male flower pistillode | 1.50E+07 | 1.10 | 98.90 |  | 85.74 | 7.20 | 5.95 |  | 20324 | 73.40 | 5.11E+06 | 33.59 | 8.92E+05 | 1.17E+05 | 9.10E+06 |
| SRX1770817 | Teratological staminate flower pistillode | 3.65E+07 | 1.14 | 98.85 |  | 82.58 | 10.20 | 6.07 |  | 21533 | 77.77 | 1.25E+07 | 33.66 | 2.21E+06 | 2.93E+05 | 2.21E+07 |
| **Average** | **-** | **2.28E+07** | **0.29** | **99.71** |  | **83.99** | **7.32** | **8.40** |  | **2.07E+04** | **7.48E+01** | **1.07E+07** | **46.08** | **1.83E+06** | **2.75E+05** | **1.05E+07** |
